# Histological and Histopathological Features of the Third Metacarpal/Tarsal Parasagittal Groove and Proximal Phalanx Sagittal Groove in Thoroughbred Horses with Racing History

**DOI:** 10.3390/ani14131942

**Published:** 2024-06-30

**Authors:** Szu-Ting Lin, Alastair K. Foote, Nicholas M. Bolas, David R. Sargan, Rachel C. Murray

**Affiliations:** 1 Department of Veterinary Medicine, University of Cambridge, Madingley Rd, Cambridge CB3 0ES, UK; linstjulie@outlook.com (S.-T.L.); drs20@cam.ac.uk (D.R.S.); 2 Rossdales Veterinary Surgeons, Cotton End Rd, Exning, Newmarket CB8 7NN, UK; alastair.foote@rossdales.com; 3 Hallmarq Veterinary Imaging, Unit 5 Bridge Park, Merrow Lane, Guildford GU4 7BF, UK; nick.bolas@hallmarq.net

**Keywords:** subchondral bone, articular cartilage, proximal phalanx sagittal groove, metacarpal/metatarsal parasagittal groove, fissure, thoroughbred

## Abstract

**Simple Summary:**

Information about the histological features of the proximal phalanx (P1) sagittal groove in racehorses is limited compared to the third metacarpal/metatarsal parasagittal groove. Fractures of the P1 sagittal groove are common in racehorses; therefore, it is important to improve the understanding of the histopathology of the P1 sagittal groove. This study aims to describe histopathological features of the third metacarpal/metatarsal parasagittal groove and the proximal phalanx sagittal groove in Thoroughbred racehorses. For third metacarpal/metatarsal parasagittal grooves, hyaline cartilage (HC), calcified cartilage (CC), and subchondral plate and trabecular bone (SCB/TB) histopathology including SCB collapse were most severe in locations with fissures (observed in a previous study) and the palmar/plantar aspect of the parasagittal groove. For P1 sagittal grooves, SCB/TB histopathology was most severe in locations with fissures, and SCB collapse was most severe in the dorsal and middle aspects of sagittal groove. In conclusion, histopathological features of bone fatigue injury were related to fissure locations, the palmar/plantar third metacarpal/metatarsal parasagittal groove, and the dorsal P1 sagittal groove. Histopathological findings in the P1 sagittal groove may be useful for investigating the pathogenesis of P1 sagittal groove fractures.

**Abstract:**

Information regarding the histopathology of the proximal phalanx (P1) sagittal groove in racehorses is limited. Twenty-nine cadaver limbs from nine Thoroughbred racehorses in racing/race-training underwent histological examination. Histological specimens of the third metacarpal/metatarsal (MC3/MT3) parasagittal grooves and P1 sagittal grooves were graded for histopathological findings in hyaline cartilage (HC), calcified cartilage (CC), and subchondral plate and trabecular bone (SCB/TB) regions. Histopathological grades were compared between (1) fissure and non-fissure locations observed in a previous study and (2) dorsal, middle, and palmar/plantar aspects. (1) HC, CC, and SCB/TB grades were more severe in fissure than non-fissure locations in the MC3/MT3 parasagittal groove (*p* < 0.001). SCB/TB grades were more severe in fissure than non-fissure locations in the P1 sagittal groove (*p* < 0.001). (2) HC, CC, and SCB/TB grades including SCB collapse were more severe in the palmar/plantar than the middle aspect of the MC3/MT3 parasagittal groove (*p* < 0.001). SCB/TB grades including SCB collapse were more severe in the dorsal and middle than the palmar/plantar aspect of the P1 sagittal groove (*p* < 0.001). Histopathology in the SCB/TB region including bone fatigue injury was related to fissure locations, the palmar/plantar MC3/MT3 parasagittal groove, and the dorsal P1 sagittal groove.

## 1. Introduction

Third metacarpal/metatarsal (MC3/MT3) parasagittal groove fracture (parasagittal fracture) and proximal phalanx (P1) sagittal groove fracture (sagittal fracture) are the most common fractures in Thoroughbred racehorses [1,2,3,4]. The risk factors [1,5,6,7], pathogenesis [8,9,10,11], and macroscopic findings [8,9,10,12,13,14] of parasagittal fractures have been well reported in previous studies. Parasagittal fractures in racehorses occur in predictable locations and are considered to be stress fractures [8,9,10,11,14,15]. It has been suggested that stress fracture occurs as subchondral bone adaptation fails [8,9,10,11,15]. Previous microscopic studies found that microcracks in the calcified cartilage and subchondral bone accumulated during repetitive bone injuries from high-speed exercises, and the coalescing and propagation of microcracks could be a predisposing factor in the development of stress fracture [8,9,10,11,15]. Pathological changes in linear defects in the calcified cartilage and subchondral bone (defined as fissures in this study) have also been observed in the MC3/MT3 parasagittal groove at locations where fractures originate [10,12,13,16]; therefore, it has been suggested that fissures could be one of the prodromal pathologies of stress fractures [9,10,12,13,16].

A sagittal fracture of P1 used to be considered as resulting from a single acute traumatic event, but imaging studies on racehorses and sport horses have shown that sagittal fractures may also develop from fatigue injuries [17,18,19,20,21,22,23,24]. Imaging studies suggested that the aetiology of sagittal fractures may be similar to parasagittal fractures, developing from failure of functional adaptation in subchondral bone and the propagation of microcracks into stress fractures [17,18,19,20]. Therefore, fissures were also suggested to be one of the prodromal pathologies of sagittal fractures [18]. Histological studies are needed to assess the relation between imaging findings of fissures and fatigue injuries [17]. However, there are no reports of histopathological findings in the proximal phalanx sagittal groove to the authors’ knowledge, except for a microcomputed tomography study reporting the adaptation of subchondral bone to race training including increased subchondral bone thickness and volumetric bone mineral density [25].

The objectives of this study were to describe the histopathological features of the MC3/MT3 parasagittal groove and P1 sagittal groove in Thoroughbred racehorses using the following two approaches:

- A comparison between locations with and without fissures in the MC3/MT3 parasagittal groove and P1 sagittal groove detected in a previous study [26]. In the previous study, fissures were identified on both cone-beam and fan-beam computed tomography and were confirmed as histological findings of microcracks in the calcified cartilage and subchondral bone plate [26]. The previous study assessed histopathological features associated with fissures regardless of MC3/MT3 or P1 origin [26]. In this study, fissures of MC3/MT3 or P1 origin were described separately.
- A comparison between dorsal, middle, and palmar/plantar aspects of the MC3/MT3 parasagittal groove and P1 sagittal groove.

It was hypothesised that (1) the histopathological features of locations with fissures would be similar between the MC3/MT3 parasagittal groove and P1 sagittal groove and (2) predilection sites of parasagittal fractures and sagittal fractures including the palmar/plantar aspect of the MC3/MT3 parasagittal groove and the dorsal and middle aspects of the P1 sagittal groove would be more associated with histopathological features of stress/fatigue injuries.

## 2. Materials and Methods

### 2.1. Materials

The study was approved by the University of Cambridge Ethical Review committee (project number: CR558).

**Figure 1:**
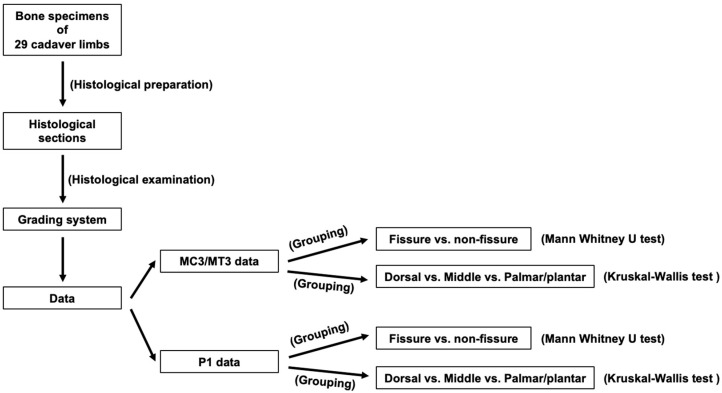
Schematic overview of the study including histological preparation, examination, grading, and data analysis. The grouping of fissures was defined in the previously published study, where fissures were identified on both cone-beam and fan-beam computed tomography and were confirmed by histological examination [26].

Twenty-nine cadaver limbs (fifteen forelimbs and fourteen hindlimbs) from 9 Thoroughbred horses (6 geldings, 2 colts, and 1 filly) aged 2–11 years were used in this study. The cadaver limbs were convenience samples from a previous study [26]. All horses had racing or race-training history. Limbs were sourced entirely from the UK where Thoroughbred racehorses are trained and raced in both directions (clockwise and anticlockwise), with training taking place off the racecourse. All horses were euthanised for reasons other than the study: 5 due to fractures (P1 fractures, medial condylar fracture, lateral condylar fracture, and proximal sesamoid bone fracture in other limbs) and 4 for reasons other than fracture (severe osteoarthritis, severe osteochondral lesion, head injury, and pelvic injury). The cadaver limbs from horses with fractures were not entirely excluded from the study, but locations affected by complete fractures were excluded from histological examination. See Figure 1.

Cadaveric limbs were collected at the carpus or tarsus level within 8 h of euthanasia and stored frozen at −20 °C. The limbs were thawed at room temperature for 24 h before post-mortem examination. 

### 2.2. Histological Preparation

**Figure 2:**
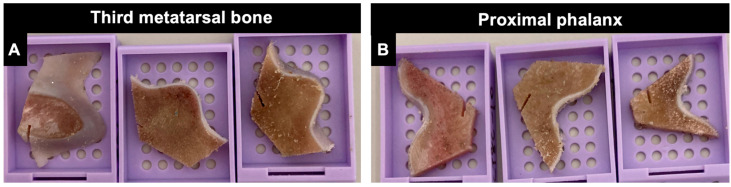
Bone specimens from a hindlimb. From left to right: bone specimens collected from the dorsal, middle, and plantar aspects of the third metatarsal parasagittal groove (**A**) and proximal phalanx sagittal groove (**B**). The lateral side of the specimen was marked using a bandsaw. The shape of specimens from the third metacarpal bone is different from specimens from the proximal phalanx.

**Figure 3:**
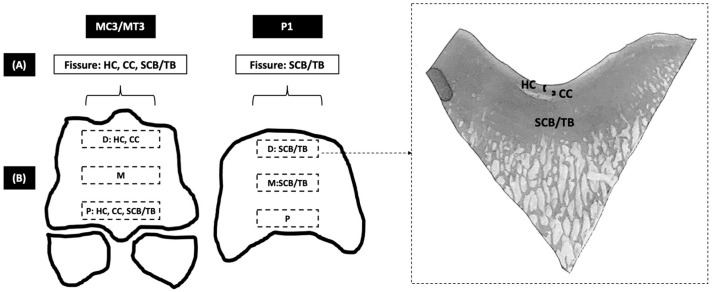
Illustration of the regions graded for histopathological changes, grouped for comparison, and outcome. Histological specimen in the box on the right demonstrated regions of hyaline cartilage (HC), calcified cartilage (CC), and subchondral plate and trabecular bone regions (SCB/TB). Histopathological grades from third metacarpal/metatarsal (MC3/MT3) and proximal phalanx (P1) were analysed separately and were compared using two approaches: (**A**) fissure and non-fissure locations and (**B**) dorsal (D), middle (M), and palmar/plantar (P) aspects. HC, CC, and SCB grades were most severe in fissure locations and the palmar/plantar aspect of the MC3/MT3 parasagittal groove. SCB grades were most severe in fissure locations and the dorsal and middle aspects of the P1 sagittal groove.

For each cadaver limb, bone specimens (4–5 mm thickness) were collected using a band saw at the dorsal, middle, and palmar/plantar aspects of the MC3/MT3 parasagittal groove and P1 sagittal groove (Figure 2 and Figure 3). Dorsal, middle, and palmar/plantar aspects were divided by the dorsal and palmar/plantar borders of the collateral fossae. Collected bone specimens were then fixed in 10% neutral buffered formalin for 7 days minimum, decalcified in hydrochloric acid for 2 days minimum, and paraffin embedded. Histological sections (4–5 μm thickness) were stained with haematoxylin and eosin (H&E) and toluidine blue.

### 2.3. Histological Examination

Histological sections were graded for histopathological changes using a previously published grading system by a trained interpreter (PhD student) and overseen by recognised specialists (Alastair K. Foote and Rachel C. Murray) [27]. The interpreter was not aware of the location (dorsal, middle, and palmar/plantar) or the identity of the histological sections. However, fissures and the origin of sections from either a third metacarpal/metatarsal bone or a proximal phalanx could not be blinded due to the concurrent presence of fissures with histopathological changes and the shape of sections (Figure 2). 

The grading of histopathological changes included the following three regions based on a previously published grading system [27] (Figure 3):

(1) Hyaline cartilage (HC): alteration in staining for glycosaminoglycans, surface irregularity, fibrillation, thickness variation, irregular chondrocyte distribution, chondrocyte loss, and chondrocyte clustering.
(2) Calcified cartilage (CC): tidemark incongruence, calcified cartilage cleft, depth variation, vascular invasion, and islands of cartilage in subchondral bone plate.
(3) Subchondral plate and trabecular bone regions (SCB/TB): sclerosis, subchondral bone collapse, the replacement of cancellous bone with dense bone, trabecular thickening, replacement with osteonal bone (increased deposition of lamellar bone), increased basic multicellular units (BMUs), replacement with woven bone, and Howship’s lacunae.

For each region, histological grades ranged from 0 (no abnormality) to 3 (severe histopathology). Representative images of the grades of histopathological changes are provided in Supplementary Figures S1–S3.

### 2.4. Histological Grades Analysis

Histological grades established in the previous study were further analysed in this study [26]. Different from the previous study [26], histological grades from MC3/MT3 and P1 were analysed separately. The grading of each histopathologic specimen in the HC, CC, and SCB/TB regions and the summary (by pooling) of all grades in each region used the following two independent grouping approaches (Figure 3):

A. Grouping by the presence or absence of fissures observed in the previous study, where fissures were identified on both cone-beam and fan-beam computed tomography and were confirmed by histological examination [26]: (1) fissure location and (2) non-fissure location.
B. Grouping by dorsal, middle, and palmar/plantar aspects, where bone specimens were collected (see Histological Preparation for details): (1) dorsal aspect, (2) middle aspect, and (3) palmar/plantar aspect.

### 2.5. Data Analysis

Statistical analyses were performed using IBM SPSS Statistics 28.0.0 software. The comparison of histological grades between fissure and non-fissure locations in the MC3/MT3 parasagittal groove and P1 sagittal groove was performed using the Mann–Whitney U test. The comparison of histological grades between the dorsal, middle, and palmar/plantar aspects of the MC3/MT3 parasagittal groove and P1 sagittal groove was performed using the Kruskal–Wallis test and a post hoc Mann–Whitney U test. A *p* value < 0.05 was considered statistically significant. Differences in histopathological features between the MC3/MT3 parasagittal groove and P1 sagittal groove were described.

## 3. Results

Of the 29 cadaver limbs in this study, 1had MC3 lateral parasagittal groove complete fracture, 1 had P1 sagittal groove complete fracture, 1 had P1 sagittal groove incomplete fracture, and the remaining 26 limbs were not affected by MC3/MT3/P1 fractures.

### 3.1. Comparison between Fissure and Non-Fissure Locations in MC3/MT3 and P1 (Figure 4)

See Table 1 and Figure 5 for details of the comparison of grades of each histopathological change in the HC, CC, and SCB/TB regions and the proportion of grades in fissure and non-fissure locations.

**Figure 4:**
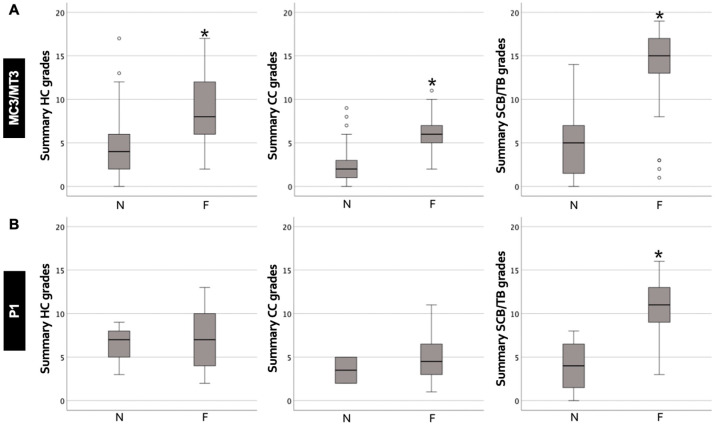
Summary of histological grades compared between fissure and non-fissure locations. HC (hyaline cartilage); CC (calcified cartilage); SCB/TB (subchondral plate and trabecular bone). (**A**) HC, CC, and SCB/TB histological grades were more severe in fissure (F) than non-fissure (N) locations in the third metacarpal/tarsal parasagittal groove (MC3/MT3). (**B**) SCB/TB histological grades were more severe in F than N locations in the proximal phalanx sagittal groove (P1). * Significant difference (*p* < 0.001). Circles above or below the end of the whiskers represented outliers.

**Figure 5:**
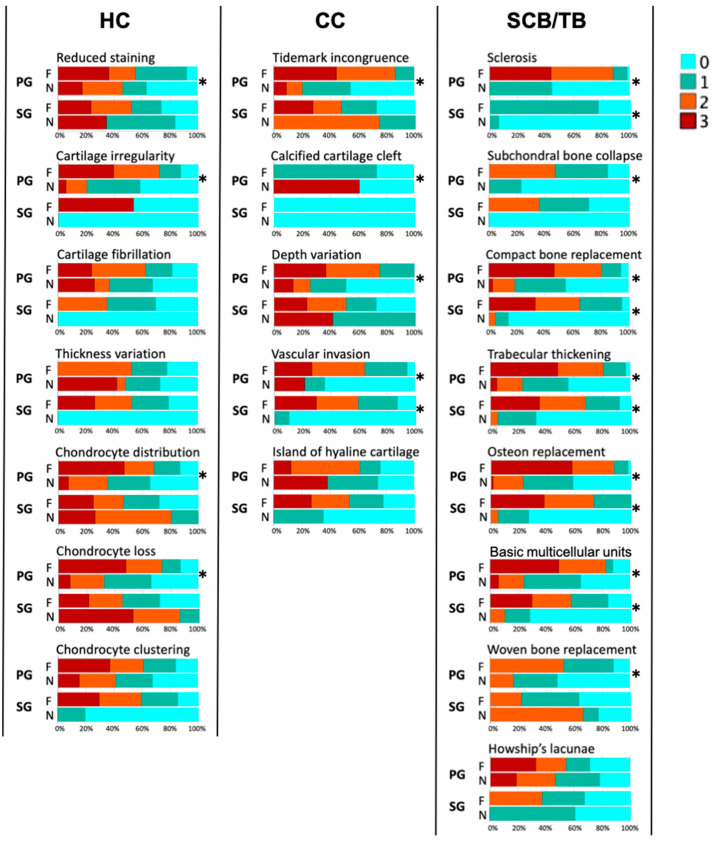
Proportion of grades for fissure and non-fissure locations. HC (hyaline cartilage); CC (calcified cartilage); SCB/TB (subchondral plate and trabecular bone); PG (third metacarpal/metatarsal parasagittal groove); SG (proximal phalanx sagittal groove); F (fissure location); N (non-fissure location). * Significant difference (*p* < 0.05).

#### 3.1.1. MC3/MT3 Parasagittal Groove

The median value and interquartile range of summary of histological grades for (1) fissure locations were 8.0 ± 7.0 in the HC region (*n* = 41), 6.0 ± 2.0 in the CC region (*n* = 41), and 15.0 ± 5.0 in the SCB/TB region (*n* = 45); and (2) non-fissure locations were 4.0 ± 5.0 in the HC region (*n* = 61), 2.0 ± 2.0 in the CC region (*n* = 59), and 5.0 ± 6.0 in the SCB/TB region (*n* = 67). The summary of the HC, CC, and SCB/TB histological grades was more severe in the fissure than non-fissure locations (*p* < 0.001 for all comparisons; Figure 3). 

#### 3.1.2. P1 Sagittal Groove

The median value and interquartile range of the summary of histological grades for (1) fissure locations were 7.0 ± 6.0 in the HC region (*n* = 41), 4.5 ± 4.0 in the CC region (*n* = 40), and 11.0 ± 4.0 in the SCB/TB region (*n* = 42); and (2) non-fissure locations were 7.0 ± 4.0 in the HC region (*n* = 6), 3.5 ± 3.0 in the CC region (*n* = 6), and 4.0 ± 7.0 in the SCB/TB region (*n* = 7). The summary of the SCB/TB histological grades was more severe in fissure than non-fissure locations (*p* < 0.001), while there was no significant difference in the summaries of the HC and CC histological grades (Figure 3). 

### 3.2. Comparison between Dorsal, Middle, and Palmar/Plantar Aspects in MC3/MT3 and P1 (Figure 6)

See Table 2 and Figure 7 for details of the comparison of grades of each histopathological change in the HC, CC, and SCB/TB regions and the proportion of grades in the dorsal, middle, and palmar/plantar aspects.

**Figure 6:**
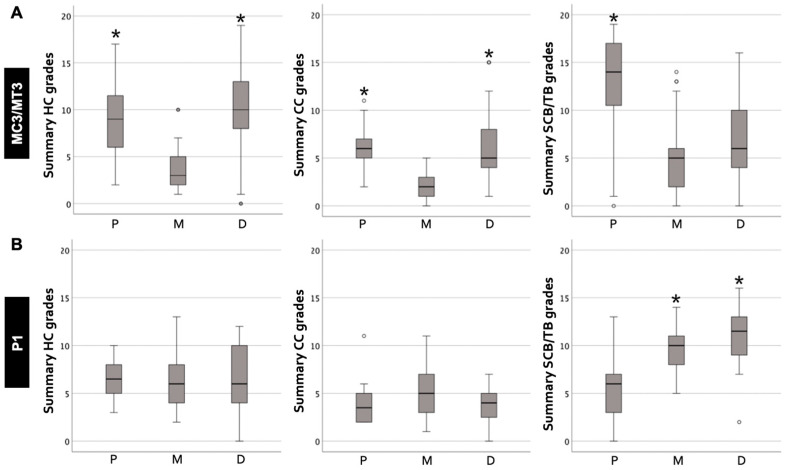
Summary of histological grades compared between dorsal, middle, and palmar/plantar aspects. HC (hyaline cartilage); CC (calcified cartilage); SCB/TB (subchondral plate and trabecular bone). (**A**) HC, CC, and SCB/TB histological grades were more severe in the palmar/plantar (P) aspect, and the HC and CC histological grades were more severe in the dorsal (D) aspect than the middle aspect (M) in the third metacarpal/tarsal parasagittal groove (MC3/MT3). (**B**) SCB/TB histological grades were more severe in the D and M aspects than the P aspect in the proximal phalanx sagittal groove (P1). * Significant difference (*p* < 0.001). Circles above or below the end of the whiskers represented outliers.

**Figure 7:**
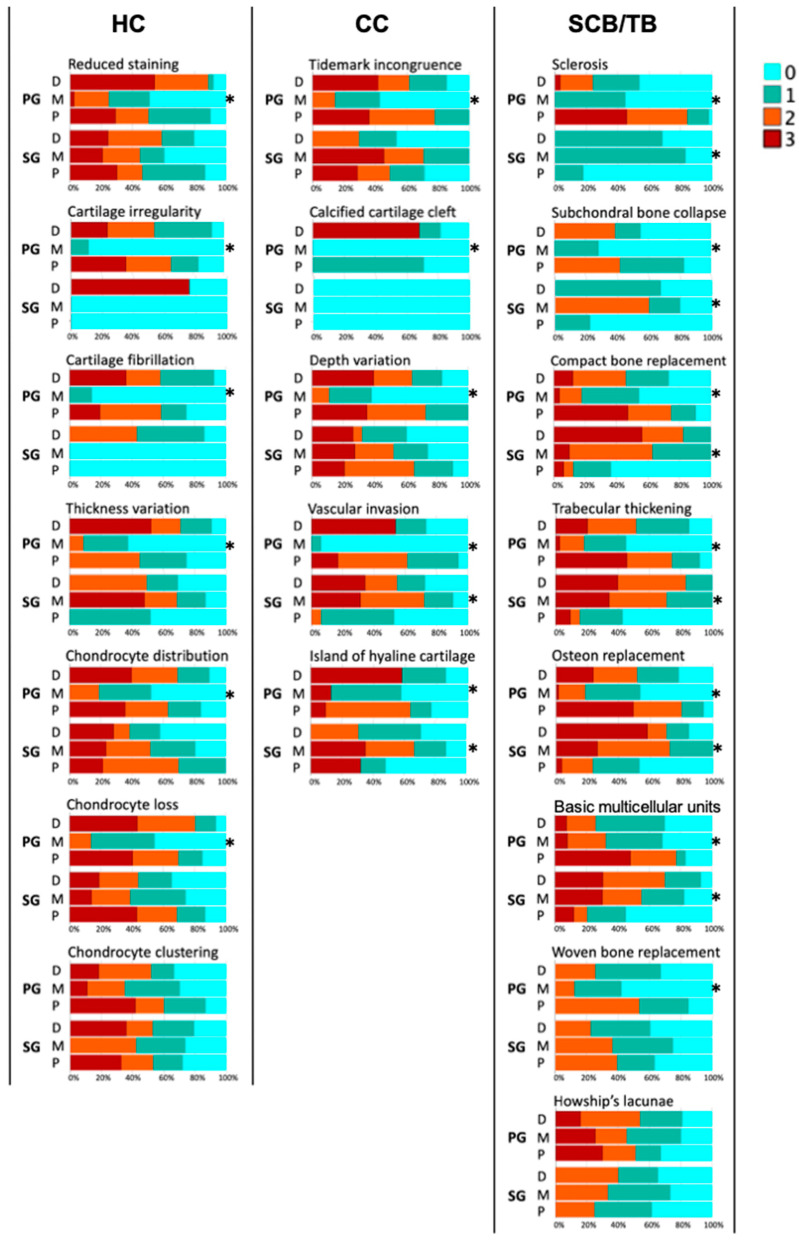
Proportion of grades for dorsal, middle, and palmar/plantar aspects. HC (hyaline cartilage); CC (calcified cartilage); SCB/TB (subchondral plate and trabecular bone); PG (third metacarpal/metatarsal parasagittal groove); SG (proximal phalanx sagittal groove); D (dorsal); M (middle); P (palmar/plantar). * Significant difference (*p* < 0.05).

#### 3.2.1. MC3/MT3 Parasagittal Groove

The median value and interquartile range of the summary of histological grades for (1) the dorsal aspect were 10.0 ± 5.0 in the HC region (*n* = 40), 5.0 ± 4.0 in the CC region (*n* = 39), and 6.0 ± 7.0 in the SCB/TB region (*n* = 41); (2) for the middle aspect were 3.0 ± 3.0 in the HC region (*n* = 48), 2.0 ± 2.0 in the CC region (*n* = 47), and 5.0 ± 5.0 in the SCB/TB region (*n* = 54); and (3) for the palmar/plantar aspect were 9.0 ± 6.0 in the HC region (*n* = 47), 6.0 ± 2.0 in the CC region (*n* = 46), and 14.0 ± 7.0 in the SCB/TB region (*n* = 56). The summaries of the HC and CC histological grades were more severe in the dorsal and palmar/plantar than the middle aspects (*p* < 0.001), and the summary of the SCB/TB histological grades was more severe in the palmar/plantar than the dorsal and middle aspects (*p* < 0.001) (Figure 3). 

#### 3.2.2. P1 Sagittal Groove

The median value and interquartile range of the summary of histological grades for (1) the dorsal aspect were 6.0 ± 6.0 in the HC region (*n* = 24), 4.0 ± 3.0 in the CC region (*n* = 23), and 11.5 ± 4.0 in the SCB/TB region (*n* = 26); (2) for the middle aspect were 6.0 ± 4.0 in the HC region (*n* = 25), 5.0 ± 5.0 in the CC region (*n* = 25), and 10.0 ± 3.0 in the SCB/TB region (*n* = 27); and (3) for the palmar/plantar aspect were 6.5 ± 3.0 in the HC region (*n* = 22), 3.5 ± 3.0 in the CC region (*n* = 22), and 6.0 ± 5.0 in the SCB/TB region (*n* = 25). The summary of the SCB/TB histological grades was more severe in the dorsal and middle than the palmar/plantar aspects (*p* < 0.001), while there was no significant difference in the summaries of the HC and CC histological grades (Figure 3).

## 4. Discussion

This study compared and described histopathological features of the MC3/MT3 parasagittal groove and P1 sagittal groove. The results of different histopathological features observed in the hyaline cartilage (HC) and calcified cartilage (CC) regions do not support our first hypothesis, that histopathological features of locations with fissures would be similar between the MC3/MT3 parasagittal groove and P1 sagittal groove. The severe histopathological changes in predilection sites of parasagittal fractures and sagittal fractures, including the palmar/plantar aspect of the MC3/MT3 parasagittal groove and the dorsal and middle aspects of the P1 sagittal groove, do support our second hypothesis that the predilection sites would be more associated with histopathological features related to fatigue injuries.

Fissure locations in the MC3/MT3 parasagittal groove were related to (1) SCB/TB changes indicating subchondral bone functional adaptation (e.g., sclerosis), bone fatigue injury (e.g., subchondral bone collapse), and initial bone repair (e.g., woven bone replacement) [8,11,15,27,28,29,30,31]; (2) CC changes indicating endochondral ossifications and functional adaptation (e.g., tidemark incongruence and vascular invasion) [8,32,33]; and (3) HC changes indicating matrix remodelling and tissue strain from strenuous exercise (e.g., reduced staining and irregular chondrocyte distribution) [15,34]. Similar SCB/TB, CC, and HC histopathological changes were also reported in fissure locations at the palmar/plantar aspect of the MC3/MT3 parasagittal groove in Thoroughbred racehorses [8,11,15]. 

Histopathological features in the palmar/plantar aspect of the MC3/MT3 parasagittal groove identified in this study corresponded to previous studies [8,11,15]. SCB/TB, CC, and HC changes were more severe in the palmar/plantar than the middle aspect. However, there was no difference in the severity of CC and HC changes between the palmar/plantar and dorsal aspects. Considering that the dorsal aspect of the MC3/MT3 bone is commonly affected by traumatic impact during fetlock joint hyperextension, the severe CC and HC changes in the dorsal parasagittal groove could reflect osteoarthritic changes [35,36,37]. In contrast, the severe SCB/TB changes in the palmar/plantar parasagittal groove indicated a stronger relationship with subchondral bone functional adaptation and fatigue injuries.

The histopathological features of fissure locations in the P1 sagittal groove were clearly different from the MC3/MT3 parasagittal groove in this study, particularly in the HC and CC regions. The finding that there was no HC histopathology associated with fissure locations may explain the previously reported lack of observation of pre-existing articular cartilage damage in proximal phalanx sagittal groove fractures as subchondral bone pathology could be hidden underneath the normal articular cartilage [38]. Vascular invasion was the only CC change associated with fissure locations and was known to represent endochondral ossifications in the progression of osteoarthritis [8,32,33]. In contrast to the HC and CC regions, SCB/TB changes indicating subchondral bone functional adaptation and bone fatigue injury were related to fissure locations in the P1 sagittal groove [8,11,15,27], which were similar to findings in the MC3/MT3 parasagittal groove. Considering the small number of non-fissure locations available for comparison in this study, care must be taken not to over-interpret the results of comparison.

SCB/TB changes in the dorsal and middle aspects of the P1 sagittal groove indicated subchondral bone functional adaptation, corresponding to the previously reported finding of increased subchondral bone thickness at the dorsal and middle aspects of the P1 sagittal groove in racehorses [25]. Furthermore, histopathological indications of fatigue injuries were also seen in the dorsal P1 sagittal, corresponding to the imaging finding that the dorsal aspect of the P1 sagittal groove is the most common predilection site of sagittal fractures [20,39]. 

In both the MC3/MT3 parasagittal groove and P1 sagittal groove, there are concurrent findings of vascular invasion in the CC region and increased BMU and subchondral bone collapse in the SCB/TB region. These findings correspond to the scenario proposed in the previous studies that bone remodelling (e.g., osteoclast activation and bone resorption in response to microdamage) were related to cross-talk between subchondral bone and articular cartilage at the osteochondral junction (e.g., angiogenesis), and once the microdamage accumulates and exceeds the capacity of self-repairing, bone failure (e.g., subchondral bone collapse) and fatigue fracture could occur [32,33,40,41,42,43,44]. The result that these histopathologies were all associated with fissures and predilection sites of stress fractures in both MC3/MT3 and P1 in this study could add further support to the theory of bone maladaptation.

The different histopathological features observed between the MC3/MT3 parasagittal groove and P1 sagittal groove, particularly in the HC and CC regions, might be related to the difference in pressure distribution during loading [45,46]. It was reported that the biomechanical properties of articular cartilage such as glycosaminoglycan content and collagen level varied depending on the consistency of loading and range of shear strain [45]. It was also found that different from the middle aspect of distal MC3, which was subjected to both compressive and shear strains, the middle aspect of proximal P1 was mainly subjected to shear strains [46]. The components of articular cartilage were not analysed in this study; further investigation may be needed to clarify the effect of biomechanics on cartilage histopathology in MC3/MT3 and P1.

In the P1 sagittal groove, the SCB/TB histopathological findings from our study indicated that subchondral and trabecular bone sclerosis, bone remodelling, and failure of functional adaptation of bone were associated with both fissure locations and predilection sites of P1 sagittal groove fracture. These findings support clinical experience of bone failure at this location in horses undertaking high-intensity training and the previous suggestion that pathology may be related to repetitive loading [17,18,21,25,47]. These results support previous descriptions of clinical patients reporting marked subchondral bone thickening on radiographic images and increased fluid signal around the P1 sagittal groove on magnetic resonance imaging as potential prodromal diagnostic imaging features of increased risk of bone failure at this location [17,18,19,20,48]. The findings from our study may therefore be useful to add weight to the value of imaging in decision-making for these type of cases [17,20,48].

Limitations of this study included the small number of cadaver limbs from a small number of horses. Also, multiple bone specimens collected from individual limbs (a maximum number of nine specimens from a limb) and horses could result in clustering, and the histopathological features may not be representative for the entire Thoroughbred population. However, the newly reported histopathological findings in the P1 sagittal groove in Thoroughbred racehorses provided valuable information that has been lacking and may be useful as a comparison for future investigation on the pathogenesis of P1 sagittal groove fractures or fissures. Also, the histopathological findings in the MC3/MT3 parasagittal groove corresponded to previous histopathological studies, strengthening the association between the palmar/plantar MC3/MT3 parasagittal groove fissures and the failure of subchondral bone functional adaptation. Data from forelimbs and hindlimbs, right and left forelimbs, and medial and lateral parasagittal grooves were pooled together and were not analysed separately to avoid leaving a small number or even no samples remaining in a stratified group. However, the authors were aware that condylar and P1 fractures occur most commonly in forelimbs and lateral parasagittal grooves, and active osseous pathology in the P1 sagittal groove was more common in hindlimbs than forelimbs [1,2,3,12,49,50]. The small number of non-fissure locations in the P1 sagittal groove limited the interpretation of the histopathological features of fissure locations. However, histopathological findings at the dorsal and middle aspects of the P1 sagittal groove may partly support the relationship between the predilection site of P1 sagittal groove fracture and the failure of subchondral bone functional adaptation. Although histopathological changes indicating bone fatigue such as subchondral bone collapse were assessed in this study, microdamage was not assessable due to the lack of basic fuchsin staining. As an alternative, the increased occurrence of BMU was assessed based on previous studies reporting the concurrent finding of BMU with microdamage, but the limitation that BMU can also represent normal bone remodelling is fully appreciated [51,52,53]. The histological grading in this study was conducted by a single observer, and the data would be more robust if two individual gradings could be established. Finally, the study only included Thoroughbred horses that had been in racing or race-training, and not all cadaver limbs had clinically relevant lameness or disease. A future study with control Thoroughbred horses and cadaver limbs is needed to validate the physiological and/or pathological background of the histopathological changes identified in this study.

## 5. Conclusions

In conclusion, fissures in the MC3/MT3 parasagittal groove and P1 sagittal groove could have similar pathogenesis to bone fatigue injuries, albeit with some differences at the cartilage level. Predilection sites of MC3/MT3 parasagittal fracture and P1 sagittal fracture had histopathology consistent with the failure of subchondral bone adaptation being involved in the pathogenesis. 

## Figures and Tables

**Table 1:** Significance of differences in histopathological grades compared between fissure and non-fissure locations. MC3/MT3: third metacarpal/metatarsal parasagittal groove. P1: proximal phalanx sagittal groove. Numbers presented in the table are *p* values.

Items	MC3/MT3	P1
Hyaline cartilage		
Reduced staining for glycosaminoglycans in cartilage	0.001 *	0.70
Cartilage surface irregularity	<0.001 **	0.86
Cartilage fibrillation	0.25	0.86
Cartilage thickness variation	0.13	0.88
Irregular chondrocyte distribution	<0.001 **	0.50
Chondrocyte loss/necrosis	<0.001 **	0.15
Chondrocyte clustering	0.13	0.08
Summary of hyaline cartilage	<0.001 **	0.82
Calcified cartilage		
Tidemark incongruence	<0.001 **	0.36
Calcified cartilage cleft	0.003 *	1.0
Calcified cartilage depth variation	<0.001 **	0.71
Vascular invasion	<0.001 **	<0.001 **
Islands of hyaline cartilage in subchondral bone plate	0.07	0.38
Summary of calcified cartilage	<0.001 **	0.17
Subchondral plate and trabecular bone		
Sclerosis of subchondral bone plate and adjacent cancellous bone	<0.001 **	0.004 *
Subchondral bone collapse	<0.001 **	0.24
Replacement of cancellous bone with dense bone	<0.001 **	<0.001 **
Trabecular thickening with reduced marrow spaces	<0.001 **	<0.001 **
Replacement with osteonal/lamellar bone	<0.001 **	<0.001 **
Increased basic multicellular units	<0.001 **	0.02 *
Replacement with woven bone	<0.001 **	0.83
Howship’s lacunae with/without osteoclast	0.40	0.77
Summary of subchondral plate and trabecular bone	<0.001 **	<0.001 **

* Significant difference (fissure > non-fissure location). ** Highly significant difference (fissure > non-fissure location).

**Table 2:** Significance of differences in histopathological grades compared between dorsal, middle, and palmar/plantar aspects. MC3/MT3: third metacarpal/metatarsal parasagittal groove. P1: proximal phalanx sagittal groove. D: dorsal. M: middle. P: palmar/plantar. Numbers presented in the table are *p* values.

Items	MC3/MT3	Post Hoc	P1	Post Hoc
Hyaline cartilage				
Reduced staining for glycosaminoglycans in cartilage	<0.001 **	D > M P > M	0.36	-
Cartilage surface irregularity	<0.001 **	D > M P > M	0.14	-
Cartilage fibrillation	<0.001 **	D > M D > P	0.14	-
Cartilage thickness variation	<0.001 **	D > M P > M	0.07	-
Irregular chondrocyte distribution	<0.001 **	D > M P > M	0.62	-
Chondrocyte loss/necrosis	<0.001 **	D > M P > M	0.15	-
Chondrocyte clustering	0.10	-	0.90	-
Summary of hyaline cartilage	<0.001**	D > M P > M	0.70	-
Calcified cartilage				
Tidemark incongruence	<0.001 **	D > M P > M	0.52	-
Calcified cartilage cleft	0.005 *	D > M P > M	1.0	-
Calcified cartilage depth variation	<0.001 **	D > M P > M	0.20	-
Vascular invasion	<0.001 **	D > M P > M P > D	0.003 *	M > P
Islands of hyaline cartilage in subchondral bone plate	<0.001 **	D > M D > P	0.04 *	M > P
Summary of calcified cartilage	<0.001 **	D > M P > M	0.06	-
Subchondral plate and trabecular bone				
Sclerosis of subchondral bone plate and adjacent cancellous bone	0.000 **	P > M P > D	<0.001**	D > P M > P
Subchondral bone collapse	<0.001 **	P > M P > D	0.04 *	D > P
Replacement of cancellous bone with dense bone	<0.001 **	P > M P > D	<0.001 **	D > P M > P
Trabecular thickening with reduced marrow spaces	<0.001 **	D > M P > M	<0.001 **	D > P M > P
Replacement with osteonal/lamellar bone	<0.001 **	D > M P > M P > D	<0.001 **	D > P M > P
Increased basic multicellular units	<0.001 **	P > M P > D	<0.001 **	D > P M > P
Replacement with woven bone	<0.001 **	P > M	0.42	-
Howship’s lacunae with/without osteoclast	0.14		0.73	-
Summary of subchondral plate and trabecular bone	<0.001 **	P > M P > D	<0.001 **	D > P M > P

* Significant difference (fissure > non-fissure). ** Highly significant difference (fissure > non-fissure).

## Data Availability

The data that support the findings of this study are available on request from the corresponding author.

## Supplementary Materials

The following supporting information can be downloaded at: https://www.mdpi.com/article/10.3390/ani14131942/s1, Figure S1: Hyaline Cartilage; Figure S2: Calcified Cartilage; Figure S3: Subchondral bone plate and trabecular bone.
