# Supplementary material for: Histological and Histopathological Features of the Third Metacarpal/Tarsal Parasagittal Groove and Proximal Phalanx Sagittal Groove in Thoroughbred Horses with Racing History
Source: Animals (Basel). 2024 Jun 30;14(13):1942. doi: 10.3390/ani14131942 (PMC11240324; doi:10.3390/ani14131942)
Supplement: Supplementary file 1 [file animals-14-01942-s001.zip › Figure S1.pdf]

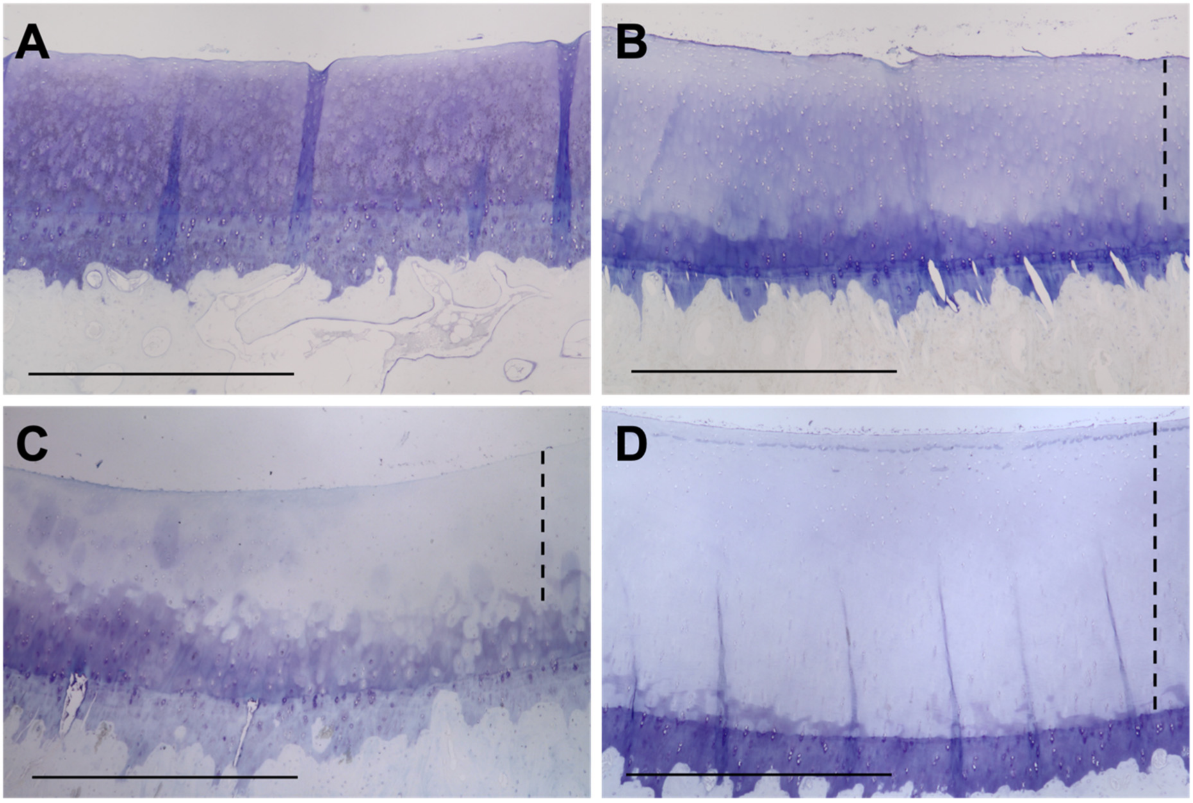

Figure S1A Hyaline cartilage assessed for reduced staining for glycosaminoglycans in cartilage. Images A – D (grade 0 to 3) were from the middle, palmar, palmar, and middle aspects of the third metacarpal/metatarsal parasagittal groove. Toluidine blue stain (A-D). Scale bar = 1 mm. (B) Diffuse, incomplete loss of stain in hyaline cartilage (dotted line). (C) Locally extensive, complete loss of stain. There were also microcracks in the calcified cartilage and subchondral bone plate in Images B and C. (D) Diffuse, complete loss of stain. There was also thickening of the hyaline cartilage layer in Image D compared to Images A – C (see scale bar).

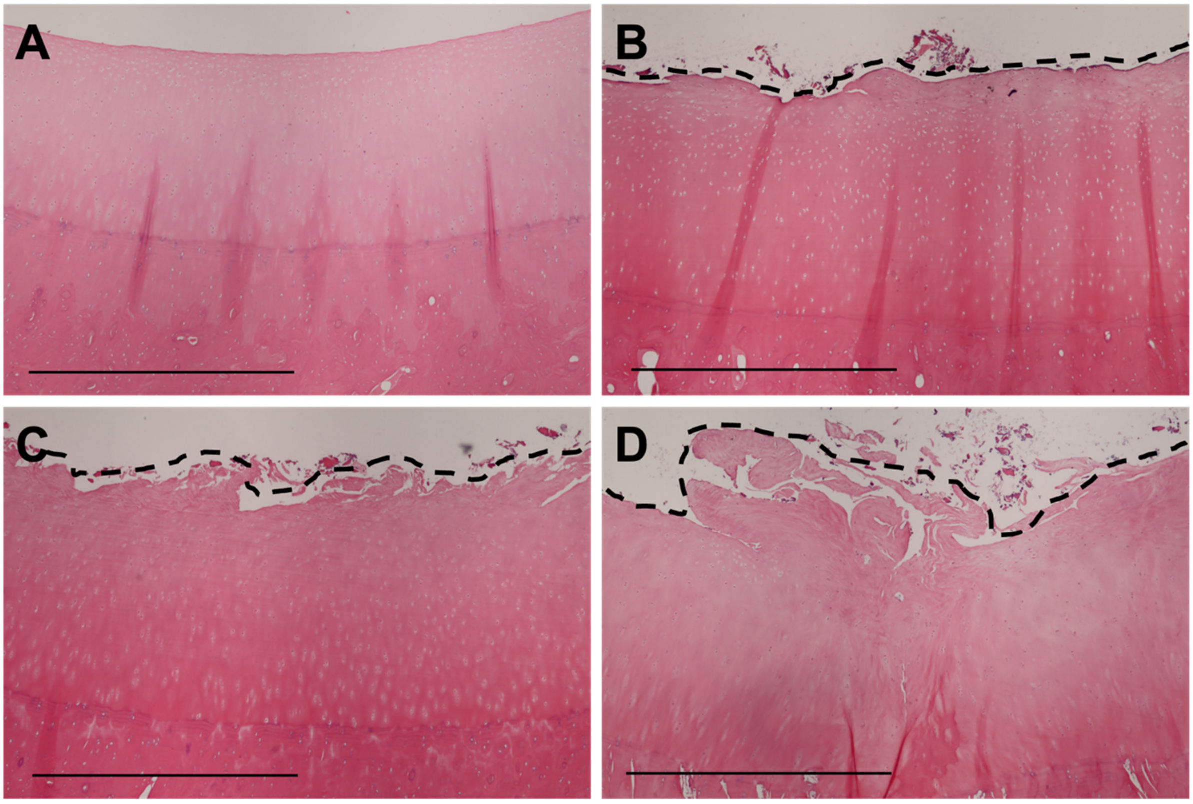

Figure S1B Hyaline cartilage assessed for cartilage surface irregularity. Images A – D (grade 0 to 3) were from the dorsal, dorsal, dorsal, and palmar aspects of the third metacarpal/metatarsal parasagittal groove. Haematoxylin and eosin (H&E) stain (A-D). Scale bar = 1 mm. (B) Mild cartilage surface irregularity (dotted line). (C) Moderate cartilage surface irregularity. (D) Severe cartilage surface irregularity.

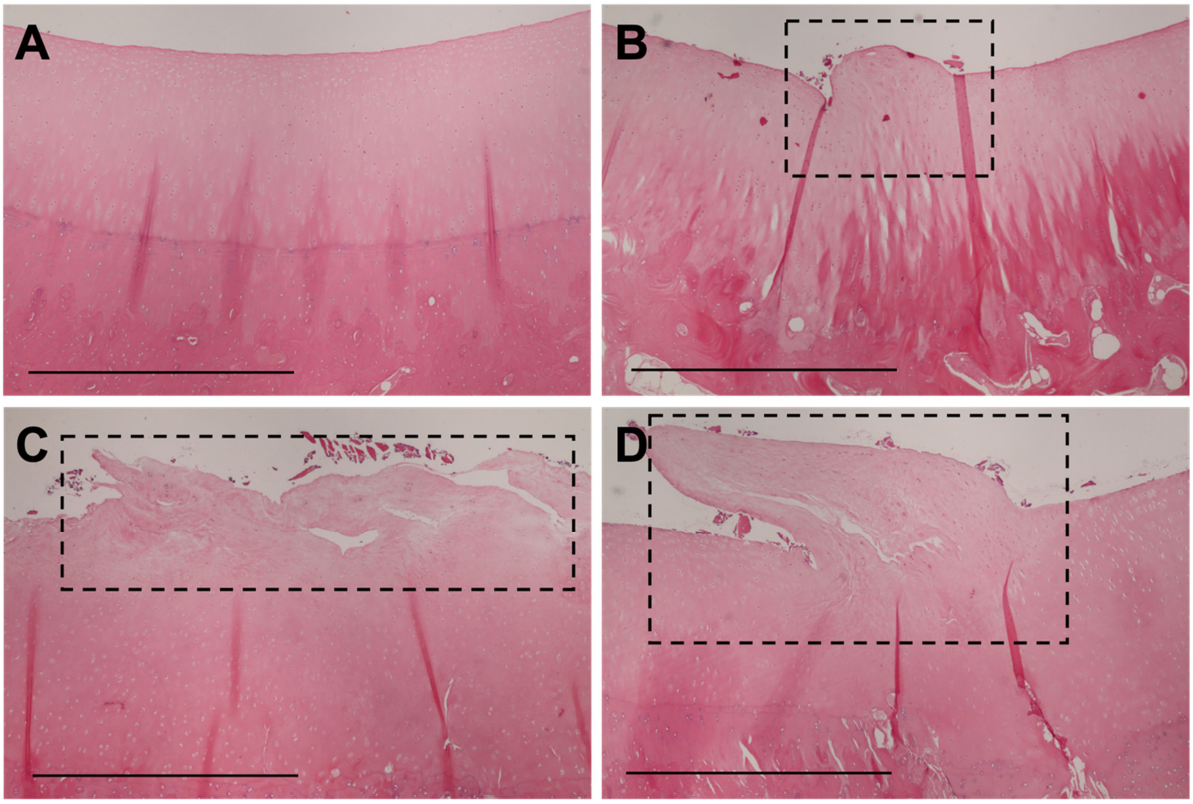

Figure S1C Hyaline cartilage assessed for cartilage fibrillation. Images A – D (grade 0 to 3) were from the dorsal, plantar, dorsal, and palmar aspects of the third metacarpal/metatarsal parasagittal groove. Haematoxylin and eosin (H&E) stain (A-D). Scale bar = 1 mm. (B) Focal, mild cartilage fibrillation (dotted line). (C) Moderate cartilage fibrillation. (D) Severe cartilage fibrillation. There were also microcracks in the calcified cartilage and subchondral bone plate in Image D.

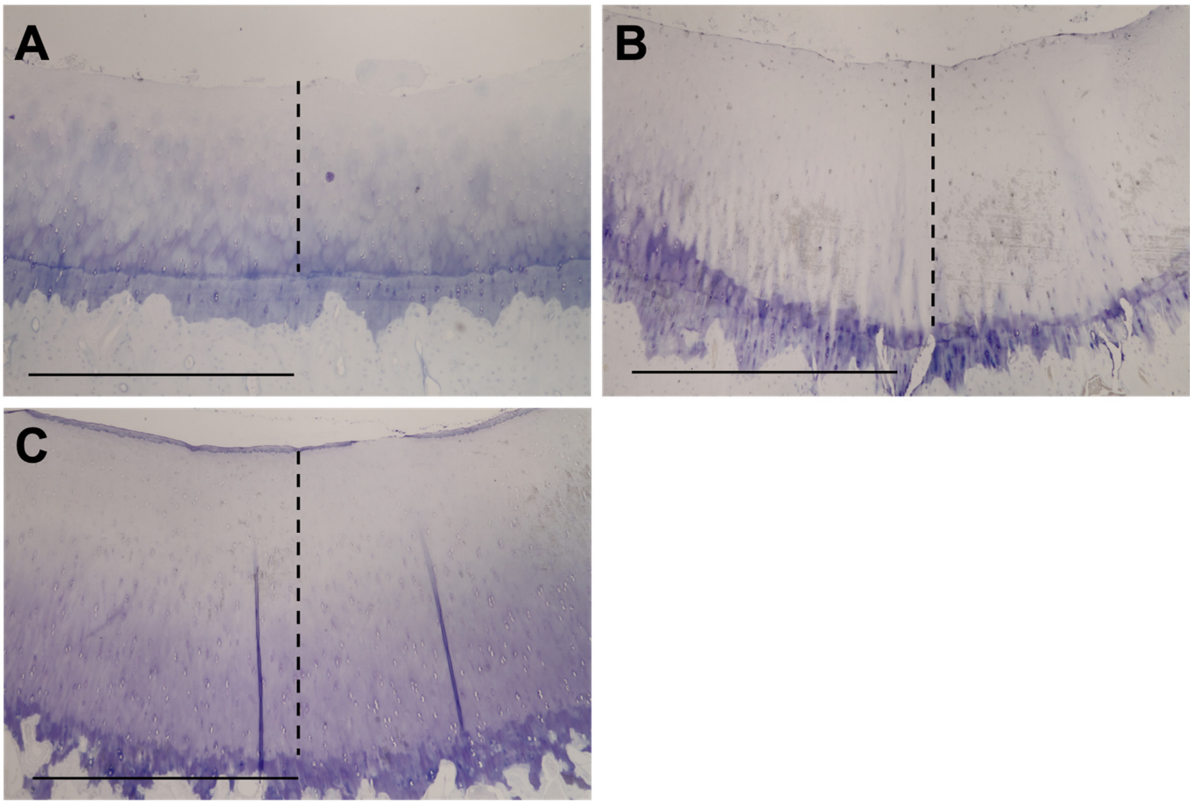

Figure S1D Hyaline cartilage assessed for cartilage thickness variation. Images A – C (grade 0 to 2) were all from the palmar, plantar, and palmar aspects of the third metacarpal/metatarsal parasagittal groove and under the same magnification. The dotted line illustrated the thickness at the sagittal line of the groove. Toluidine blue stain (A-C). Scale bar = 1 mm. (B) Mild thickening of hyaline cartilage (dotted line). There were also microcracks in the calcified cartilage and subchondral bone plate in Image B. (C) Moderate thickening of hyaline cartilage. (D) Severe thickening of hyaline cartilage. There was no grade 3 image due to lack of grade 3 changes at this location.

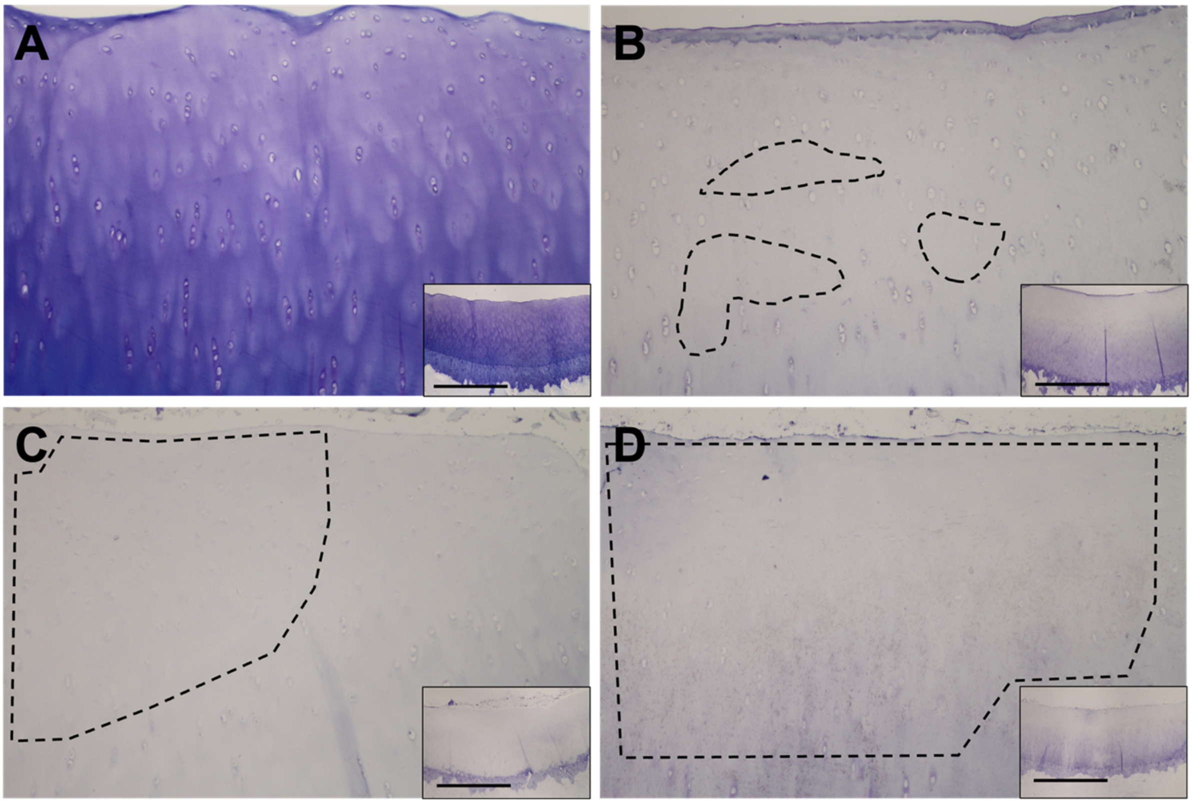

Figure S1E Hyaline cartilage assessed for irregular chondrocyte distribution. Images A – D (grade 0 to 3) were from the middle, palmar, palmar, and palmar aspects of the third metacarpal/metatarsal parasagittal groove. Toluidine blue stain (A-D). Scale bar = 1 mm. (B) Multi-focal areas with alteration in distribution (dotted line). (C) Locally extensive area with alteration in distribution. (D) Diffuse area with alteration in distribution. There was also severe reduced staining for glycosaminoglycans in the hyaline cartilage layer in Images C-D compared to Image A.

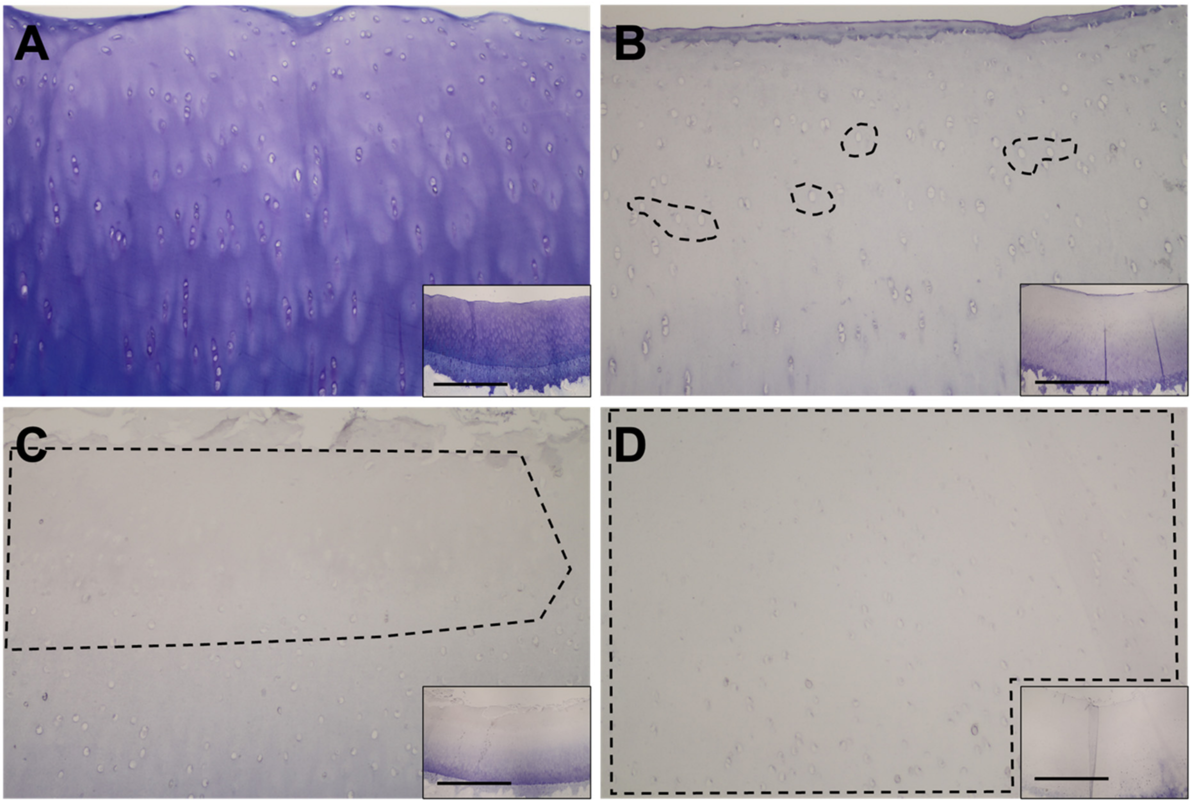

Figure S1F Hyaline cartilage assessed for chondrocyte loss/necrosis. Images A – D (grade 0 to 3) were all from the dorsal aspect of the third metacarpal/metatarsal parasagittal groove. Toluidine blue stain (A-D). Scale bar = 1 mm. (B) Mild, focal areas with empty lacunae (dotted line). (C) Moderate loss of chondrocytes. (D) Severe loss of chondrocytes. There was also severe reduced staining for glycosaminoglycans in the hyaline cartilage layer in Images C-D compared to Image A.

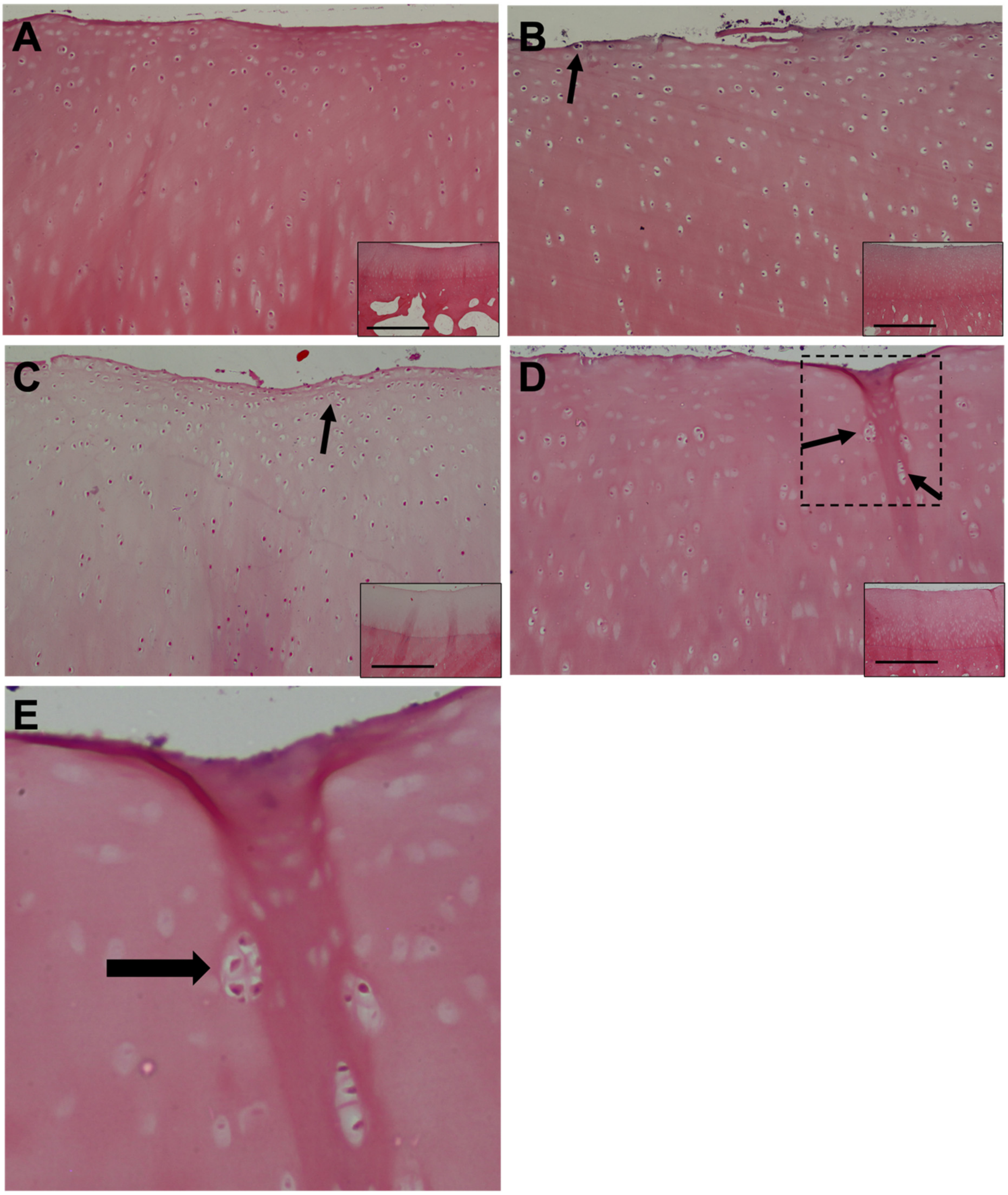

Figure S1G Hyaline cartilage assessed for chondrocyte clustering. Images A – D (grade 0 to 3) were from the middle, palmar, dorsal, and middle aspects of the third metacarpal/metatarsal parasagittal groove. Haematoxylin and eosin (H&E) stain (A-E). Scale bar = 1 mm. (B) Double chondrocytes clustering (arrow). (C) Triplet chondrocytes clustering (arrow) and increased numbers of chondrocytes. (D) Large numbers of chondrocytes clustering (arrows). Image E was magnified from the dotted area in Image D.
