# Supplementary material for: Histological and Histopathological Features of the Third Metacarpal/Tarsal Parasagittal Groove and Proximal Phalanx Sagittal Groove in Thoroughbred Horses with Racing History
Source: Animals (Basel). 2024 Jun 30;14(13):1942. doi: 10.3390/ani14131942 (PMC11240324; doi:10.3390/ani14131942)
Supplement: Supplementary file 1 [file animals-14-01942-s001.zip › Figure S2.pdf]

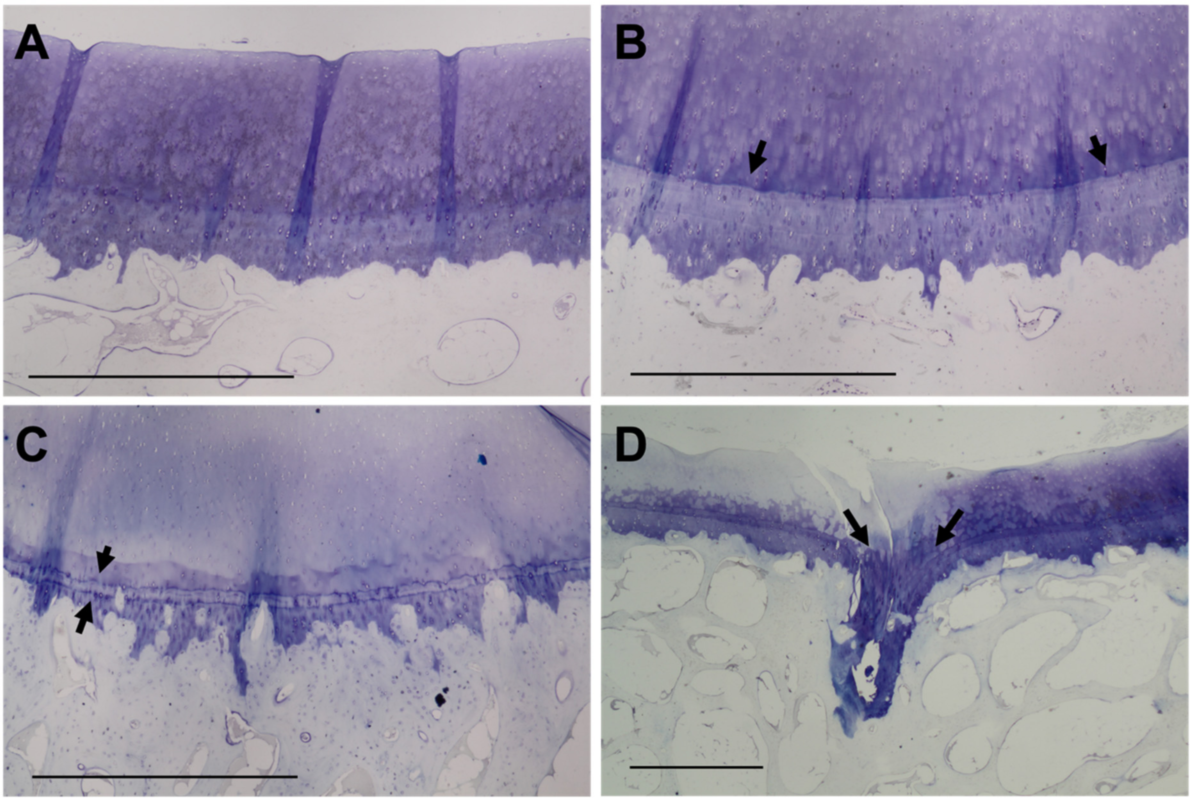

Figure S2A Calcified cartilage assessed for tidemark incongruence. Images A – D (grade 0 to 3) were from the middle, middle, dorsal, and plantar aspects of the third metacarpal/metatarsal parasagittal groove. Toluidine blue stain (A-D). Scale bar = 1 mm. (B) Irregular tidemark (arrows). (C) Tidemark duplication (arrows). (D) Absence/disruption of tidemark (arrows). The hyaline cartilage layer was collapsed and there were microcracks in the calcified cartilage and subchondral bone plate (defined as fissure) in Image D.

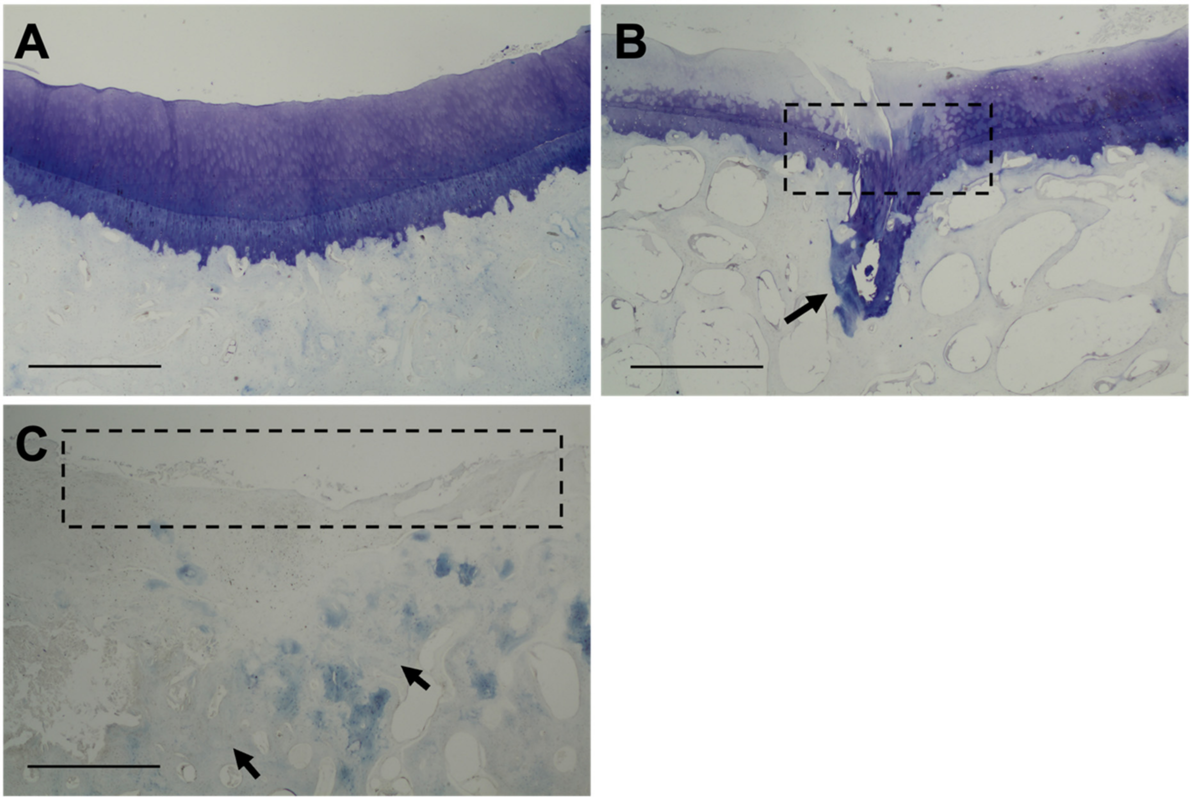

Figure S2B Calcified cartilage assessed for calcified cartilage cleft. Images A – C (grade 0, 1, and 3) were from the middle, plantar, and dorsal aspects of the third metacarpal/metatarsal parasagittal groove. Toluidine blue stain (A-C). Scale bar = 1 mm. (B) Focal cleft (dotted line). The hyaline cartilage layer was collapsed and there were microcracks in the calcified cartilage and subchondral bone plate in Image B (arrow). (C) A complete loss of hyaline and calcified cartilage exposing the underlying subchondral bone (dotted line). There was sclerosis of subchondral bone plate and cancellous bone in Image C (arrows). There was no image for grade 2 because no grade 2 calcified cartilage clefts were detected.

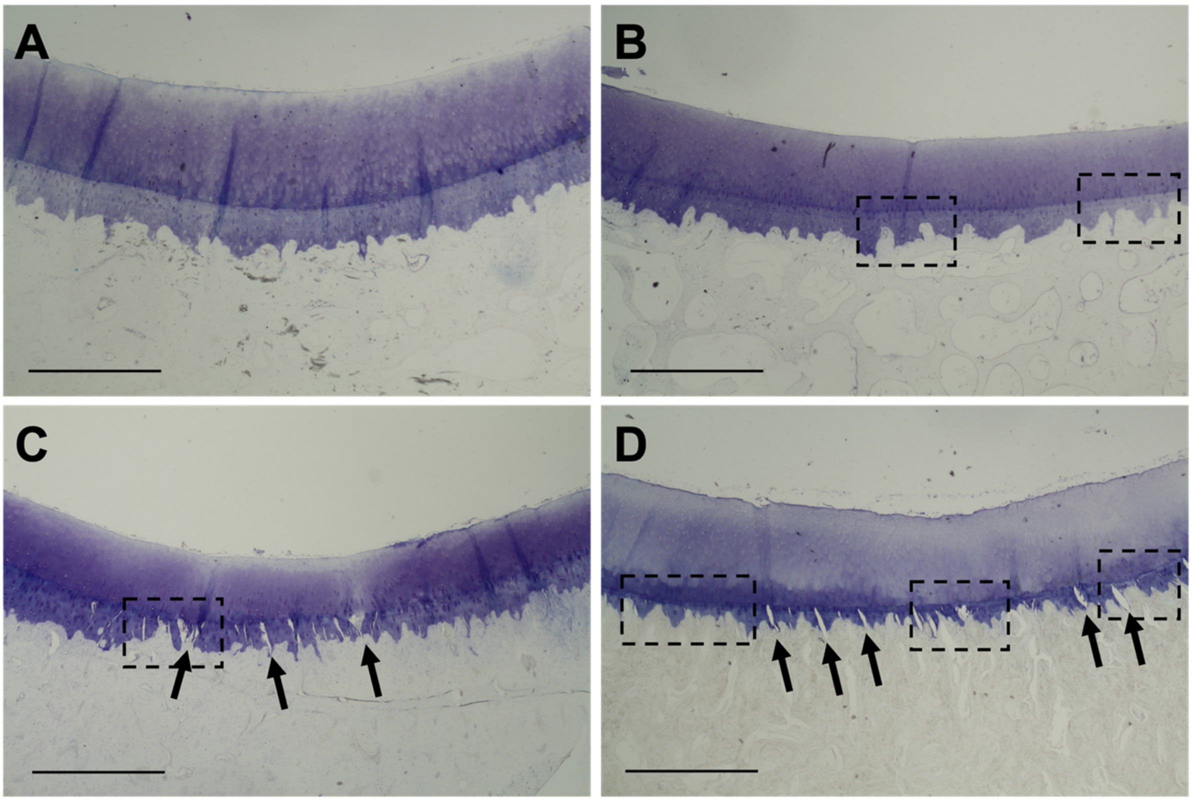

Figure S2C Calcified cartilage assessed for calcified cartilage depth variation. Images A – D (grade 0 to 3) were from the middle, middle, palmar, and palmar aspects of the third metacarpal/metatarsal parasagittal groove. Toluidine blue stain (A-D). Scale bar = 1 mm. (B) Mild variation with incomplete loss of the calcified cartilage layer (dotted line). (C) Moderate variation with focal, complete loss of the calcified cartilage layer. (D) Severe variation with diffuse, complete loss of the calcified cartilage layer. There were also microcracks in the calcified cartilage and subchondral bone plate in Images C and D (arrows).

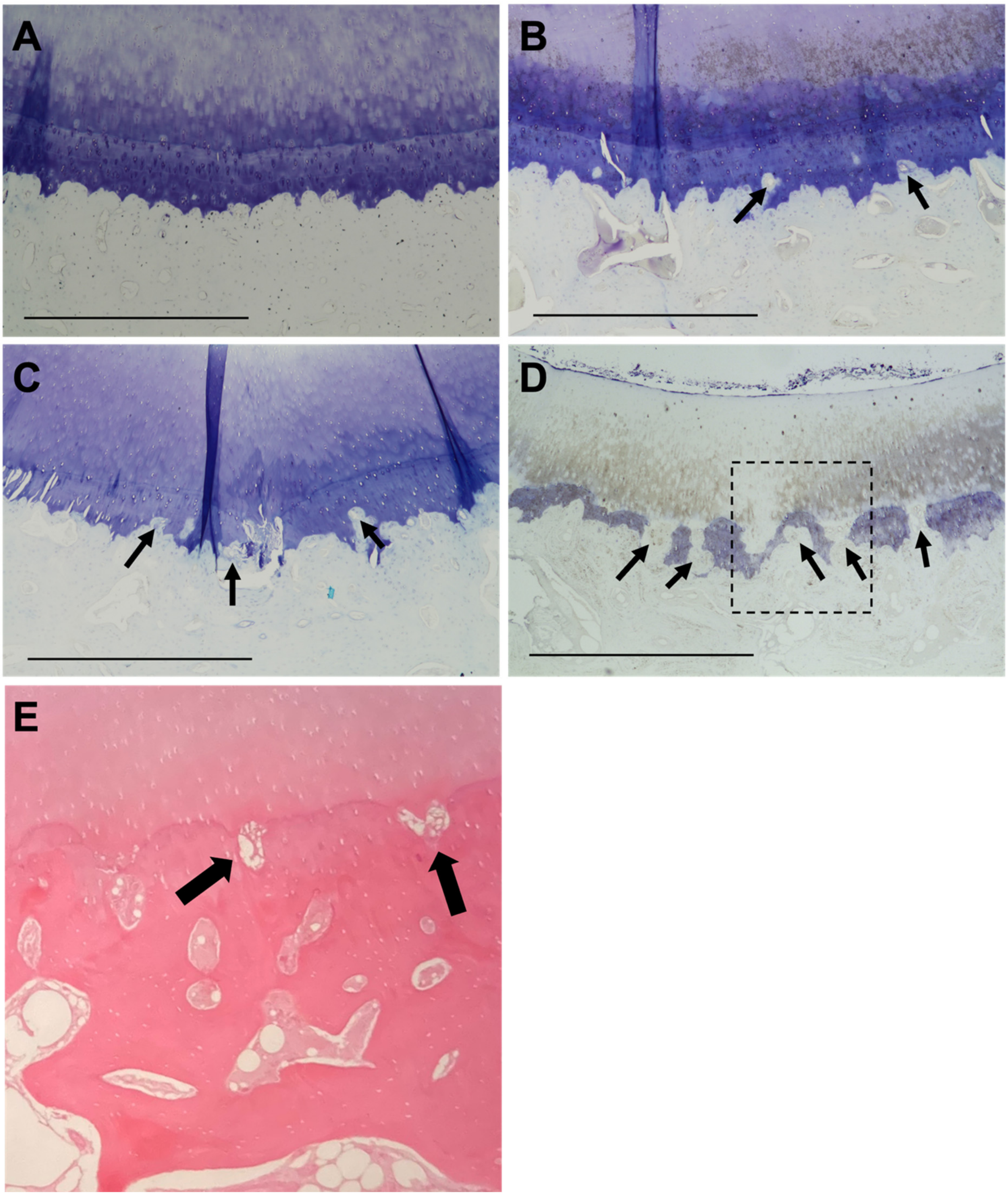

Figure S2D Calcified cartilage assessed for vascular invasion. Images A – D (grade 0 to 3) were from the middle, dorsal, middle, and middle aspects of the proximal phalanx sagittal groove. Toluidine blue (A-D) and haematoxylin and eosin (H&E) stain (E). Scale bar = 1 mm. (B) Occasional vascular invasion in the osteochondral interface (arrows). (C) Moderate vascular invasion in the osteochondral interface. (D) Frequent vascular invasion in the osteochondral interface. There were multiple bony islands with vascular channel invading through the osteochondral interface. Image E was magnified from the dotted area in image D.

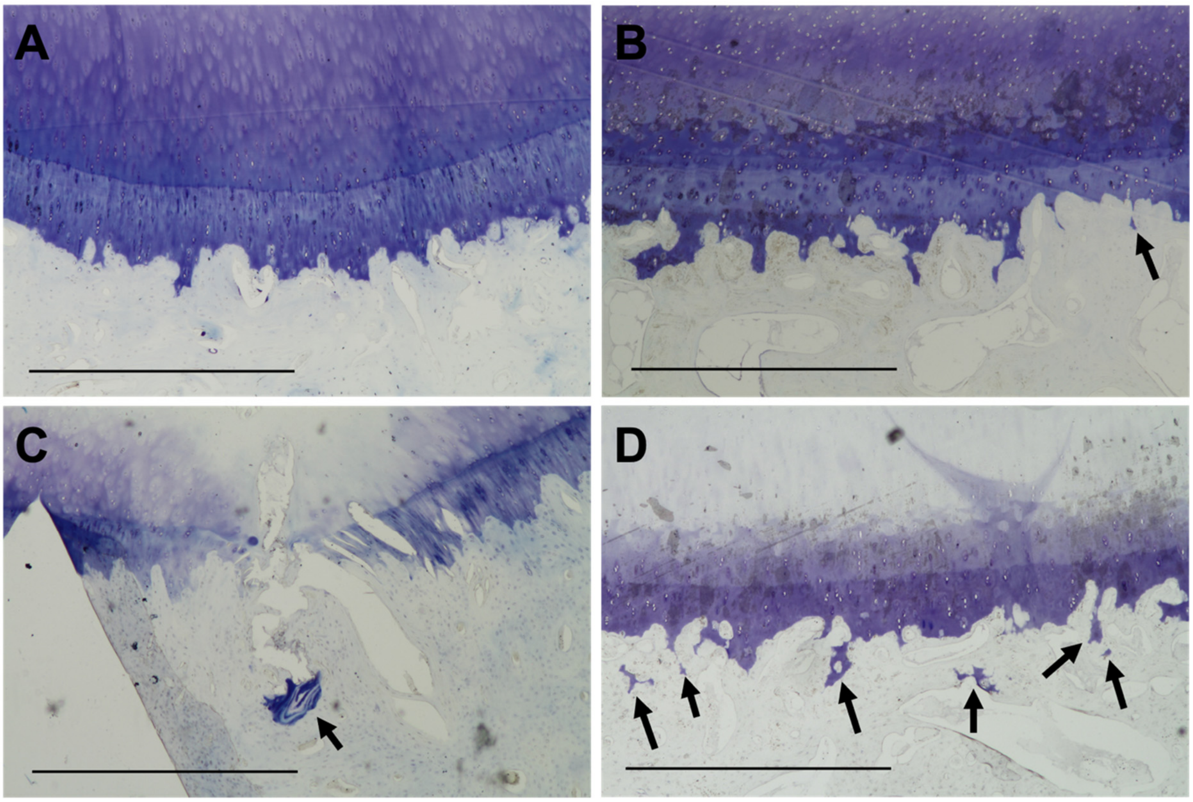

Figure S2E Calcified cartilage assessed for islands of hyaline cartilage in the subchondral bone plate. Images A – D (grade 0 to 3) were from the middle, middle, plantar, and middle aspects of the third metacarpal/metatarsal parasagittal groove. Toluidine blue stain (A-D). Scale bar = 1 mm. (B) Focal, small islands of cartilage (arrows). (C) Large, single island of cartilage. The hyaline cartilage layer was collapsed and there were microcracks in the calcified cartilage and subchondral bone plate in Image C. (D) Large numbers of islands of cartilage.
