# Supplementary material for: Histological and Histopathological Features of the Third Metacarpal/Tarsal Parasagittal Groove and Proximal Phalanx Sagittal Groove in Thoroughbred Horses with Racing History
Source: Animals (Basel). 2024 Jun 30;14(13):1942. doi: 10.3390/ani14131942 (PMC11240324; doi:10.3390/ani14131942)
Supplement: Supplementary file 1 [file animals-14-01942-s001.zip › Figure S3.pdf]

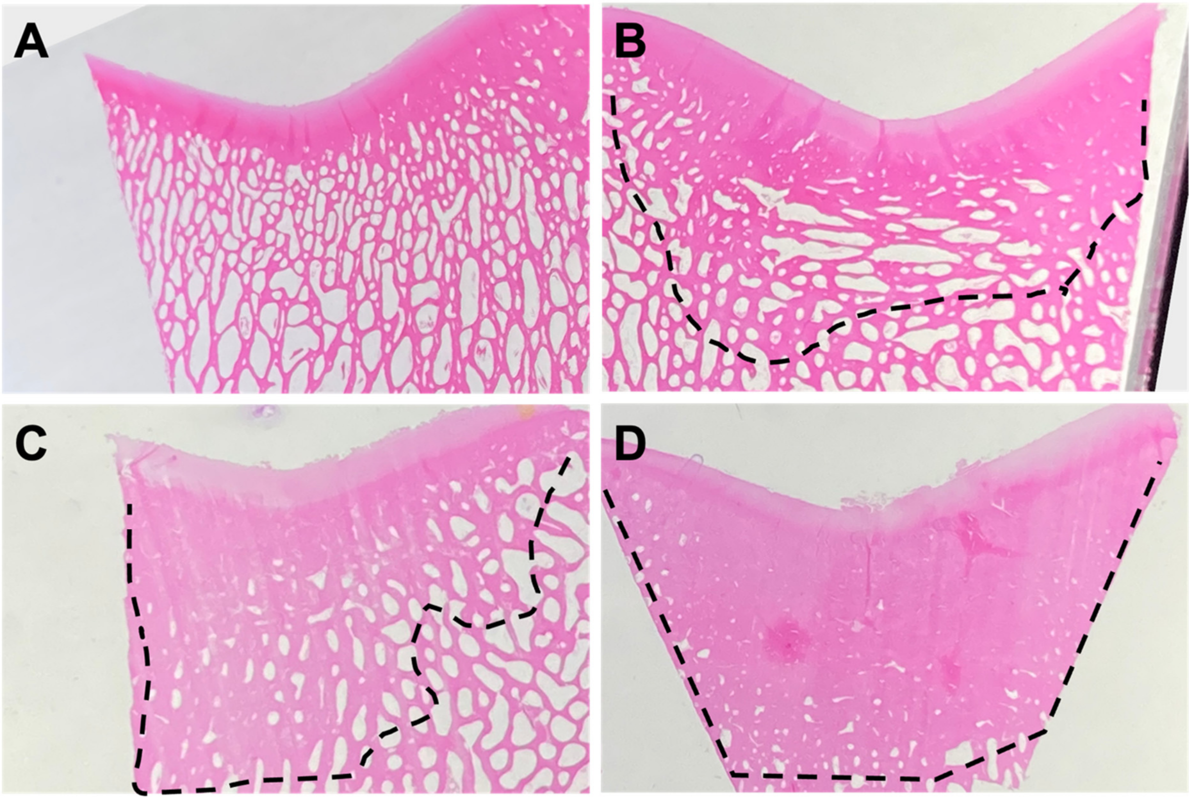

Figure S3A Subchondral and trabecular bone assessed for sclerosis. Images A – D (grade 0 to 3) were from the dorsal, plantar, palmar, and palmar aspects of the third metacarpal/metatarsal parasagittal groove. Haematoxylin and eosin (H&E) stain (A-D). (B) Mild sclerosis (dotted line). (C) Moderate sclerosis. (D) Severe sclerosis extending into non-weight-bearing area.

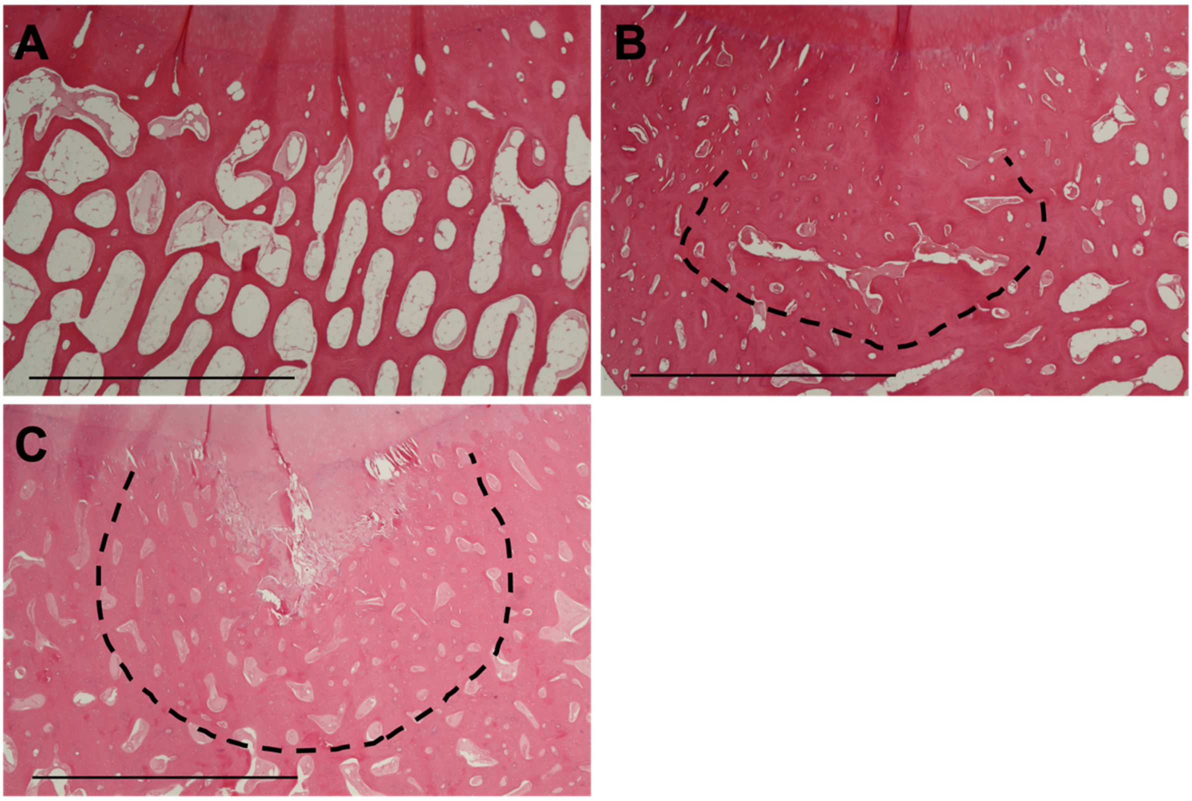

Figure S3B Subchondral and trabecular bone assessed for subchondral bone collapse. Images A – C (grade 0 to 2) were from the dorsal, palmar, and palmar aspects of the third metacarpal/metatarsal parasagittal groove. Haematoxylin and eosin (H&E) stain (A-C). Scale bar = 1 mm. (B) Small, focal area of subchondral bone collapse (dotted line). There were also microcracks in the calcified cartilage and subchondral bone plate in Images B. (C) Subchondral bone collapse with osteonecrosis. A focal calcified cartilage cleft was present, the hyaline cartilage layer was collapsed, and there were microcracks in the calcified cartilage and subchondral bone plate in Image C. There was no image for grade 3 due to lack of grade 3 subchondral bone collapse.

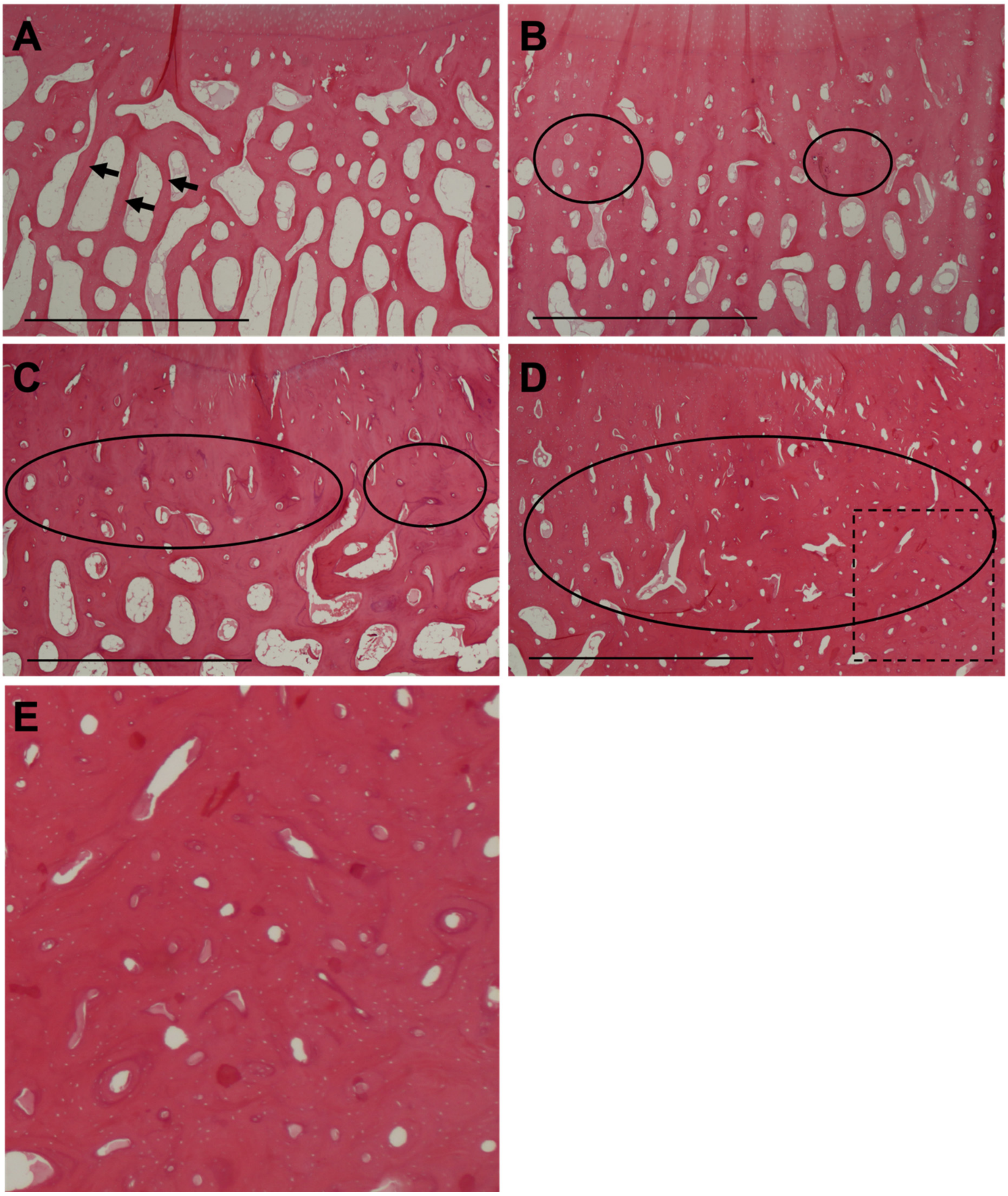

Figure S3C Subchondral and trabecular bone assessed for replacement of cancellous bone with compact bone. Images A – D (grade 0 to 3) were from the middle, dorsal, plantar, and palmar aspects of the third metacarpal/metatarsal parasagittal groove. Haematoxylin and eosin (H&E) stain (A-E). Scale bar = 1 mm. Areas normally having cancellous bone (arrows in image A) were decreased and replaced by compact bone (circled areas). (B) Mild, focal replacement (circled area). (C) Moderate replacement. (D) Severe replacement. Image E was magnified from the dotted area in image D. There was no cancellous bone remained in the area. There were also microcracks in the calcified cartilage and subchondral bone plate in Images C and D.

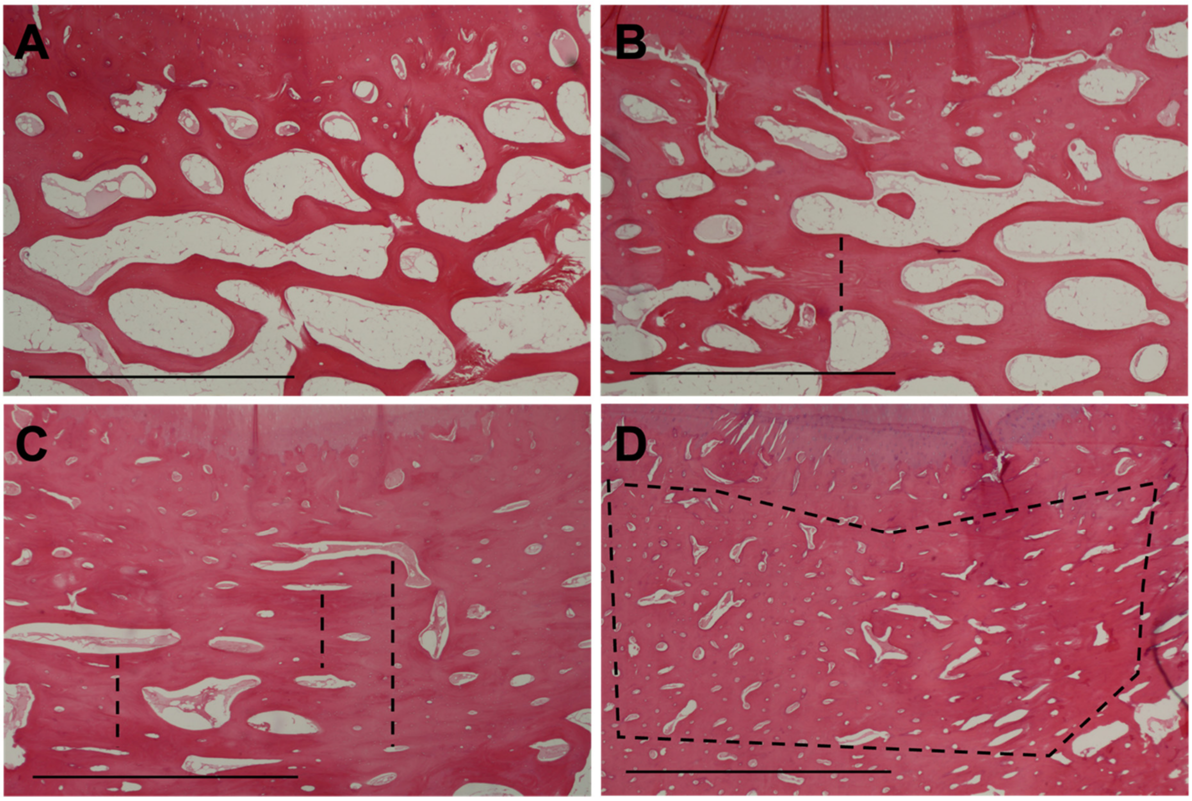

Figure S3D Subchondral and trabecular bone assessed for trabecular thickening with reduced marrow spaces. Images A – D (grade 0 to 3) were from the plantar, palmar, middle, and middle aspects of the proximal phalanx sagittal groove. Haematoxylin and eosin (H&E) stain (A-D). Scale bar = 1 mm. (B) Mild, focal trabecular thickening (dotted line). (C) Moderate trabecular thickening (dotted lines). (D) Severe, diffuse loss of marrow spaces (dotted area). There were also microcracks in the calcified cartilage and subchondral bone plate in Image D.

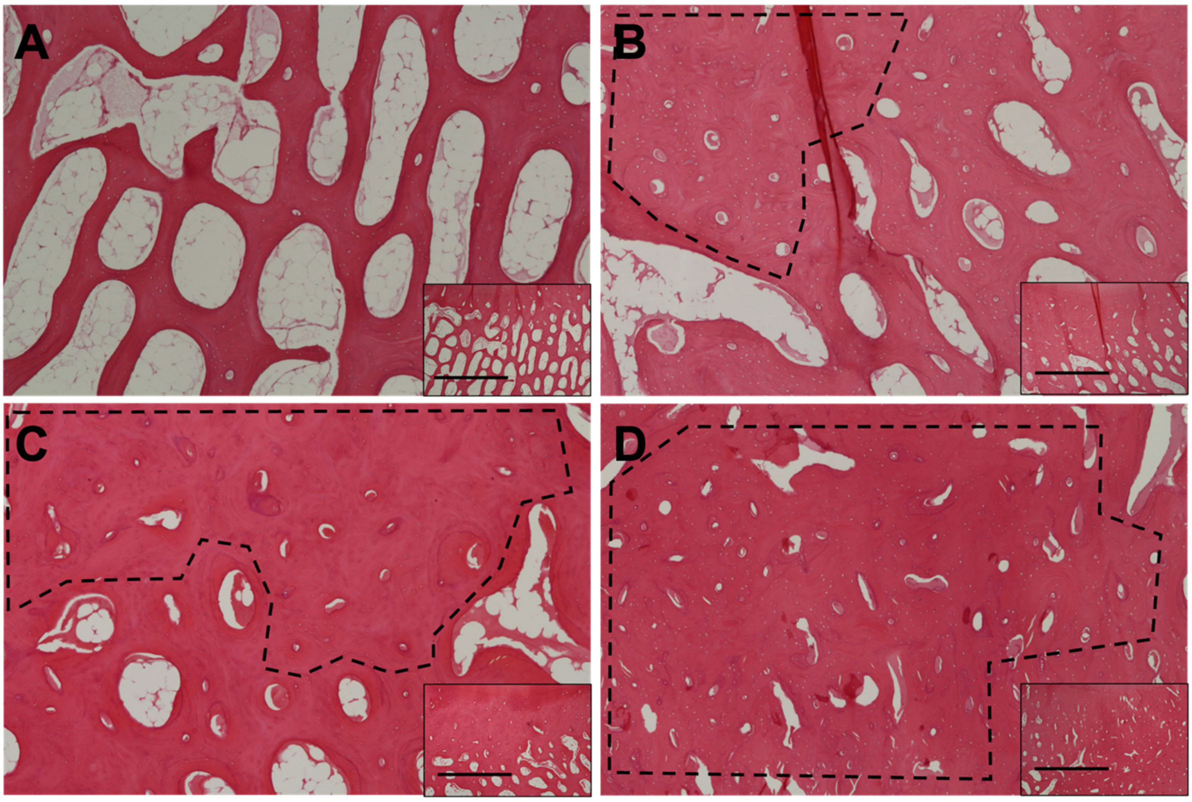

Figure S3E Subchondral and trabecular bone assessed for replacement with osteonal bone. Images A – D (grade 0 to 3) were from the dorsal, dorsal, dorsal, and palmar aspects of the third metacarpal/metatarsal parasagittal groove. Haematoxylin and eosin (H&E) stain (A-D). Scale bar = 1 mm. Areas with osteonal structure of bone (cylinders with layers of concentric lamellae) were increased (dotted lines). (B) Mild, focal replacement (dotted line). (C) Moderate, locally extensive replacement (dotted line). (D) Severe, diffuse replacement (dotted line).

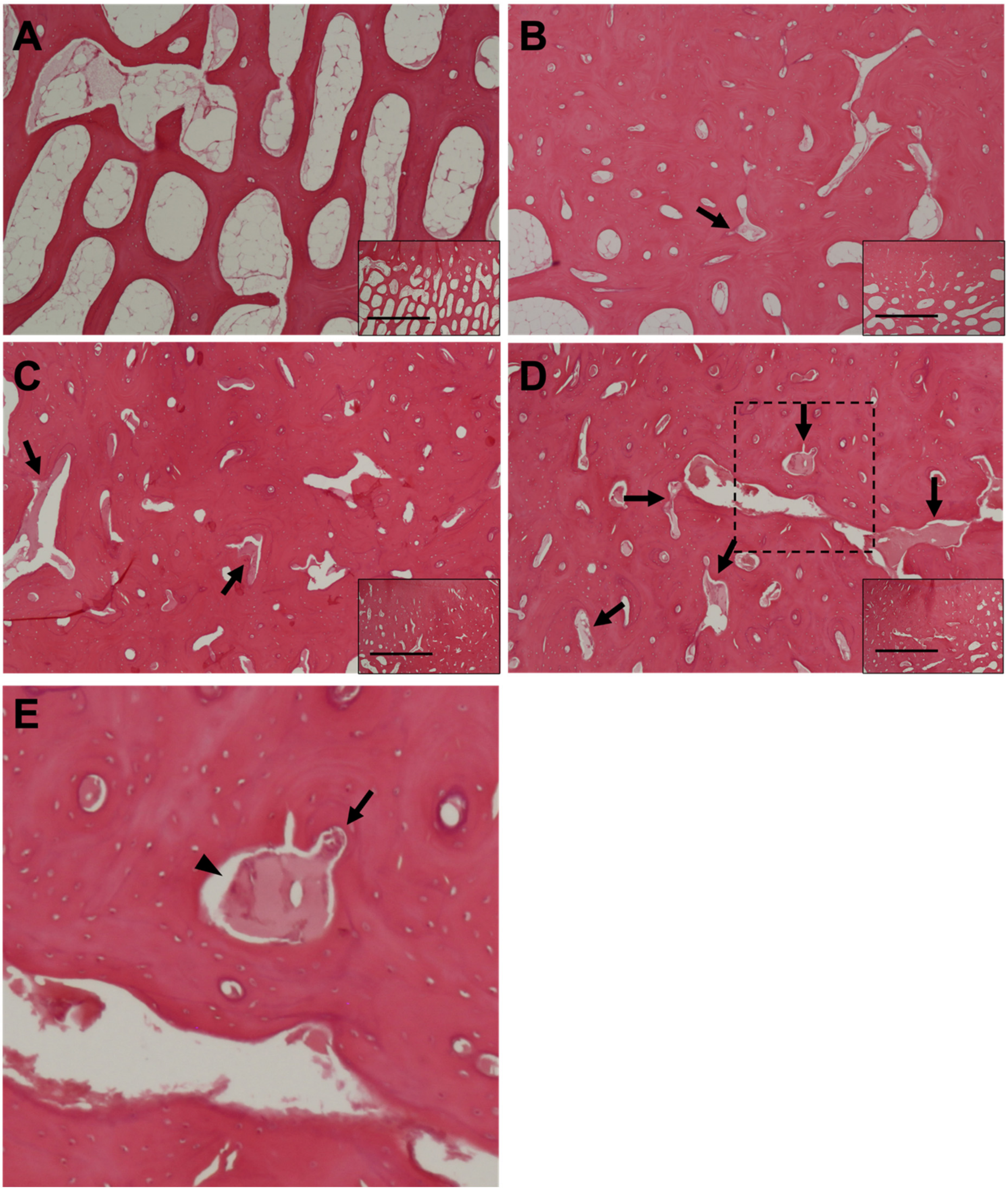

Figure S3F Subchondral and trabecular bone assessed for increased basic multicellular units (BMU). Images A – D (grade 0 to 3) were from the dorsal, middle, palmar, and palmar aspects of the third metacarpal/metatarsal parasagittal groove. Haematoxylin and eosin (H&E) stain (A-E). Scale bar = 1 mm. (B) Focal BMU (arrow). (C) Multi-focal BMU (arrows). (D) Large numbers of BMU (arrows). Image E was magnified from the dotted area in image D, showing a BMU consisting of a group of mononucleated (arrowhead) and multinucleated (arrow) cells.

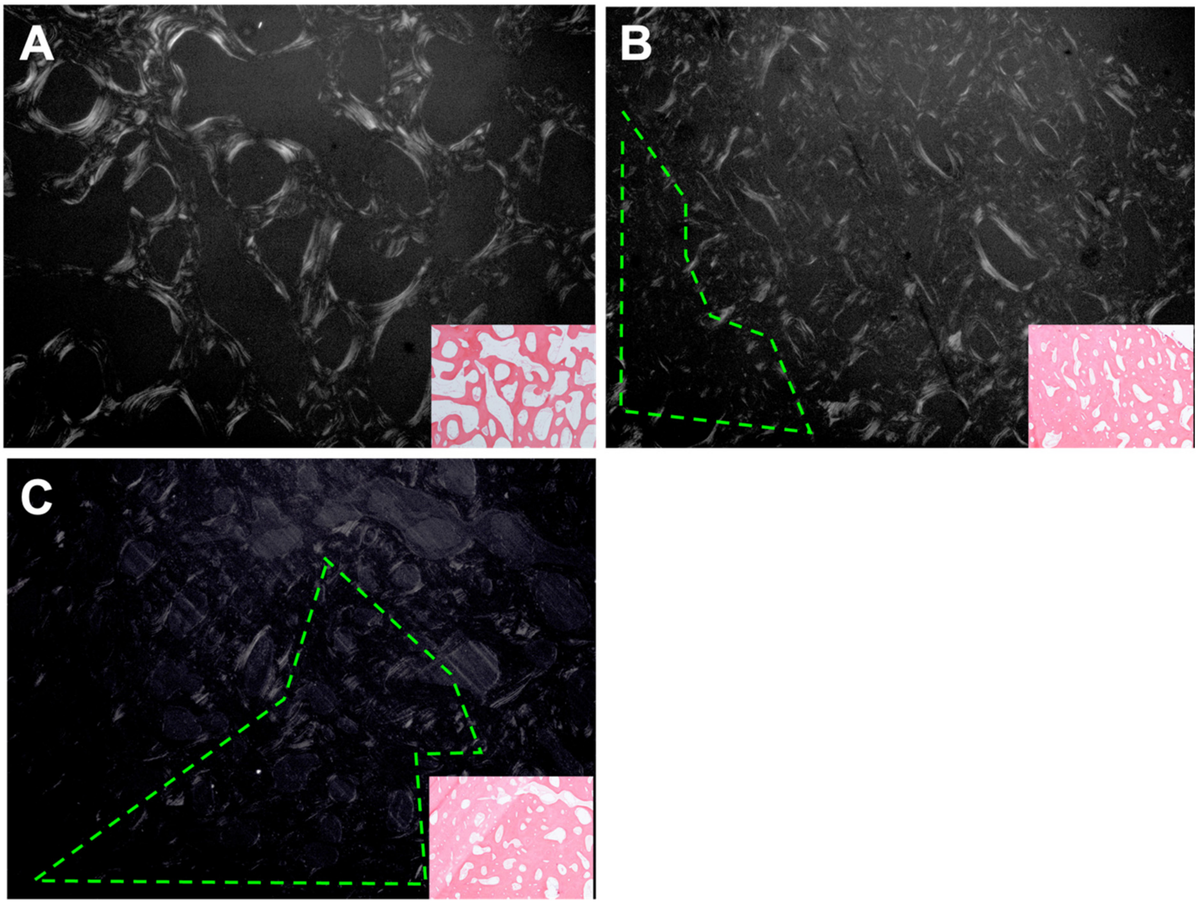

Figure S3G Subchondral and trabecular bone assessed for replacement with woven bone. Images A – D (grade 0 to 3) were from the middle, palmar, and palmar aspects of the third metacarpal/metatarsal parasagittal groove. Polarised light microscope images (A-C). Scale bar = 1 mm. (B) Focal woven bone replacement with absence of light transmission (dotted line). (C) Locally extensive woven bone replacement (dotted line). There was no image for grade 3 due to lack of grade 3 woven bone replacement.

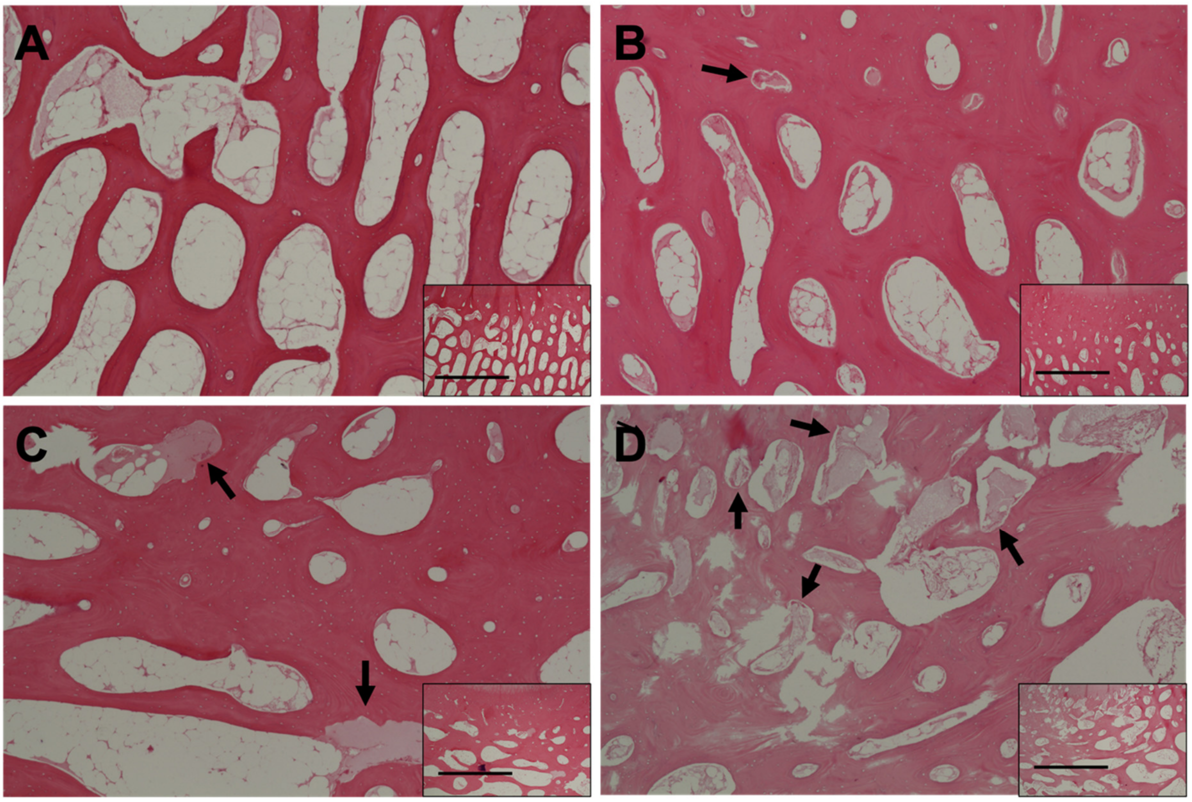

Figure S3H Subchondral and trabecular bone assessed for Howship's lacunae. Images A – D (grade 0 to 3) were from the dorsal, dorsal, middle, and plantar aspects of the third metacarpal/metatarsal parasagittal groove. Haematoxylin and eosin (H&E) stain (A-D). Scale bar = 1 mm. (B) Focal lacunae (arrow). (C) Multi-focal lacunae/bone lysis (arrows). (D) Large numbers of bone resorption (arrows).
